# Sinulolides A–H, New Cyclopentenone and Butenolide Derivatives from Soft Coral *Sinularia* sp.

**DOI:** 10.3390/md12105316

**Published:** 2014-10-23

**Authors:** Bin Yang, Xiaoyi Wei, Jingxia Huang, Xiuping Lin, Juan Liu, Shengrong Liao, Junfeng Wang, Xuefeng Zhou, Lishu Wang, Yonghong Liu

**Affiliations:** 1 Key Laboratory of Tropical Marine Bio-resources and Ecology, Guangdong Key Laboratory of Marine Materia Medica, Research Center for Marine Microbes, South China Sea Institute of Oceanology, Chinese Academy of Sciences, Guangzhou 510301, China; E-Mails: bingo525@163.com (B.Y.); xiupinglin@hotmail.com (X.L.); ljuan2010@qq.com (J.L.); ljrss@126.com (S.L.); junfeng1982a@163.com (J.W.); xfzhou@scsio.ac.cn (X.Z.); 2 Key Laboratory of Plant Resources Conservation and Sustainable Utilization, South China Botanical Garden, Chinese Academy of Sciences, Guangzhou 510650, China; E-Mail: wxy@scbg.ac.cn; 3 Zhongshan Ophthalmic Center, Sun Yat-Sen University, Guangzhou 510060, China; E-Mail: 13694217880@163.com; 4 Jilin Provincial Academy of Chinese Medicine Sciences, Changchun 130021, China; E-Mail: wls6856@163.com

**Keywords:** soft coral, *Sinularia* sp., sinulolide, NF-κB

## Abstract

Eight new compounds, sinulolides A–H (**1**–**8**), along with two known compounds, α-methoxy-2,3-dimethyl-butenolide (**9**) and sinularone D (**10**), were isolated from the soft coral *Sinularia* sp. The structures of these compounds were elucidated on the basis of extensive spectroscopic analysis. The absolute configurations were determined on the basis of electronic circular dichroism (ECD) data analysis. Compounds **5** and **10** exhibited moderate effects for the inhibition of NF-κB activation.

## 1. Introduction

**Figure 1:**
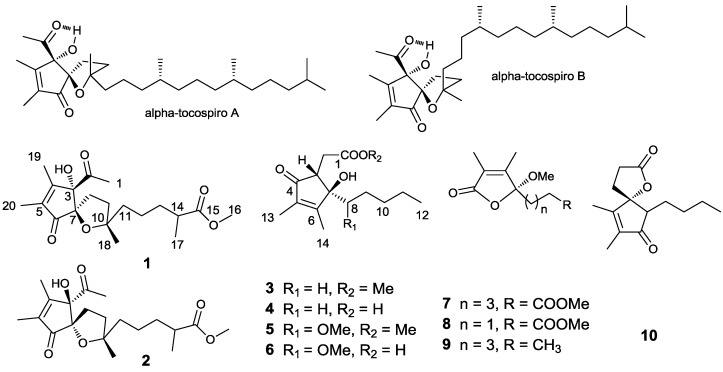
Structures of metabolites **1**–**10**.

Genus *Sinularia* is a soft coral belonging to the phylum, Cnidaria, class Alcyonaria and family Alcyoniidae. It constitutes a dominant portion of the biomass in the tropical reef environment [1]. Many bioactive metabolites, including sesquiterpenes [2,3,4], diterpenes [5,6,7,8,9] and polyhydroxylated steroids [10,11,12], have been studied, and the isolated components display a range of biological activities, such as antimicrobial, anti-inflammatory, glucose transport in rat adipocytes and cytotoxic activities [13,14,15,16,17]. During the course of our investigation on the bioactive chemical constituents from the soft coral, eight new compounds, sinulolides A–H (**1**–**8**), along with two known compounds, α-methoxy-2,3-dimethyl-butenolide (**9**) and sinularone D (**10**) (Figure 1), were isolated from *Sinularia* sp., collected off the Dongluo Island, Sanya, in July, 2009, at a depth of 10 m. The bioactivities of these compounds were determined through bioactivity tests using high-throughput screening (HTS). We describe herein the isolation, structure elucidation and bioactivities of these compounds.

## 2. Results and Discussion

**Figure 2:**
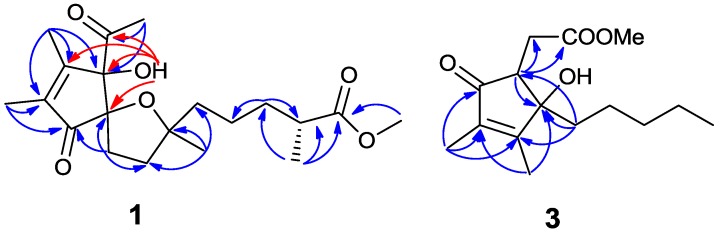
Key HMBC correlations of compounds** 1** and **3**.

**Figure 3:**
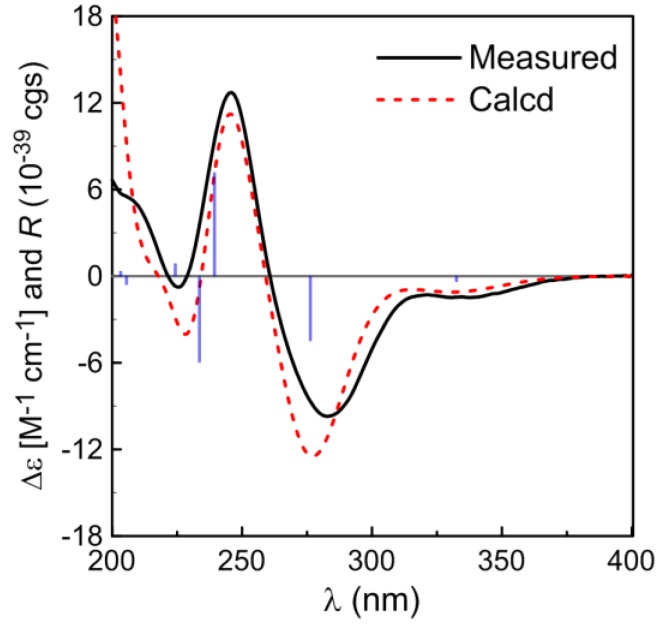
Calculated and experimental electronic circular dichroism (ECD) spectra of compound **1**.

Compound **1** was isolated as a colorless oil. Its molecular formula was assigned as C_20_H_30_O_6_ based on the HRESIMS at m/z 367.2117 [M + H]^+^, accounting for six degrees of unsaturation. The ^1^H NMR spectrum indicated the presence of one methoxyl singlet (δ_H_ 3.66, s, H-16), four methyl singlets (δ_H_ 2.01, H-1, 1.83, H-19, 1.82, H-20, 1.04, H-18), one methyl doublet (δ_H_ 1.15, t, *J* = 7.0 Hz, H-17) and a low-field exchangeable hydroxyl proton (δ_H_ 4.72, s, H-3), in addition to 11 aliphatic protons (Table 1). The ^13^C-NMR and HMQC spectra of **1** showed the presence of six methyls, five methylenes, one methine and eight quaternary carbons, including two olefinic carbons (δ_C_ 163.1, 139.5), three oxygen-bearing quaternary carbons (δ_C_ 89.1, 92.3, 86.7), two ketones (δ_C_ 207.0, 204.7) and one carbonyl (δ_C_ 177.4). The above functionalities account for four of the six degrees of unsaturation in the molecule, revealing a bicyclic structure for **1**. The ^1^H and ^13^C NMR spectra of **1 **were similar to those of *α*-tocospiro A [18,19], with the difference of the side-chain, which was confirmed by the HMBC experiment. The HMBC interactions from H_3_-16 to C-15, H-13 to C-12, C-14, C-15 and C-17, H-14 to C-12, C-13, C-15 and C-17 and H_3_-17 to C-13, C-14 and C-15 led to the connectivity of the subunits to form a linear chain (Figure 2). Subsequently, the linear side chain was determined to be linked to the nucleus at C-10 on the basis of the HMBC interactions from H_3_-18 to C-9, C-10 and C-11. For the spiro moiety, the relative stereochemistry was shown to be the same as that of α-tocospiro A on the basis of similar specific rotation and the NOESY spectrum. Circular dichroism (CD) data also support the absolute configuration of **1** to be identical to that of α-tocospiro C [20]. The absolute configuration was further determined by electronic circular dichroism (ECD) (Figure 3). The calculated ECD showed diagnostic cotton effects around 226 (positive), 246 (negative) and 287 (positive) nm, consistent with the experimental ECD. Thus, the absolute configuration was established as 3*S*, 7*R* and 10*S*, whereas the configurations at C-14 remained to be determined.

**Table 1:** ^1^H and ^13^CNMR spectroscopic data for compounds **1** and **2** (500/125 MHz, in CDCl_3_, δ in ppm, *J* in Hz).

Position	1		2	
	^13^C	^1^H	^13^C	^1^H
1	25.0	2.01 s	24.8	2.01 s
2	207.0		207.0	
3	89.1	4.72 s	89.4	4.68 s
4	163.1		163.2	
5	139.5		139.5	
6	204.7		205.0	
7	92.3		92.7	
8	36.5	1.87 m	36.8	1.88 m
		1.76 m		1.76 m
9	33.0	2.41 m	33.3	2.38 m
		1.76 m		1.76 m
10	86.7		86.9	
11	41.0	1.62 m	41.4	1.62 m
12	22.5	1.41 m	22.5	1.41 m
		1.36 m		1.36 m
13	34.4	1.67 m	34.2	1.64 m
		1.41 m		1.40 m
14	39.5	2.48 m	39.3	2.44 m
15	177.4		177.2	
16	51.5	3.66 s	51.5	3.67 s
17	17.2	1.15 d (7.0)	17.0	1.14 d (7.0)
18	25.7	1.04 s	25.4	1.29 s
19	12.0	1.83 s	11.8	1.83 s
20	8.9	1.82 s	8.7	1.81 s

**Figure 4:**
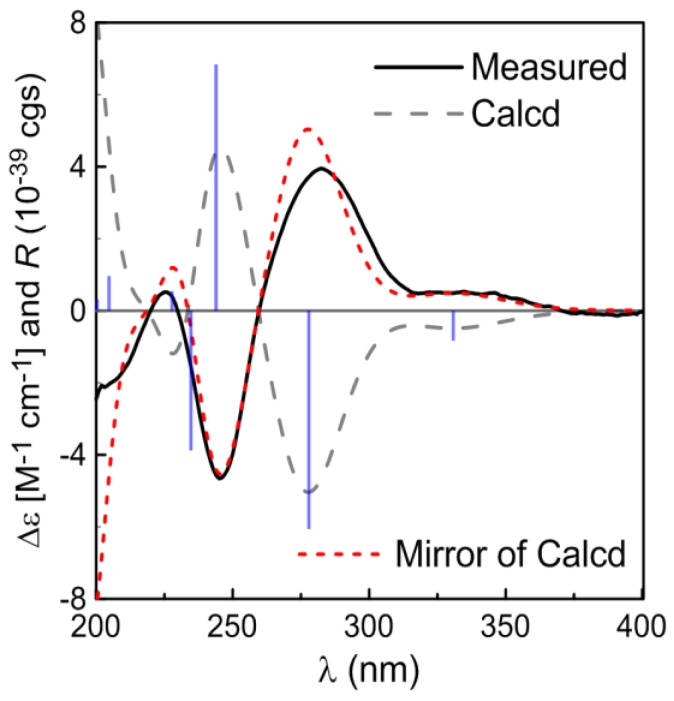
Calculated and experimental ECD spectra of compound **2**.

The NMR spectroscopic data of **1** and **2** are very similar, except for the downfield shift of CH_3_-18 (1.04→1.29) (Table 1), implying **2 **to be a C-10 epimer of **1**, as is evident from *α*-tocospiro B [18,19]. However, the optical rotation and the CD spectrum of **2** are the opposite sign to the data of **1**. The measured CD curve of **2 **was very similar to the calculated ECD for 3*R*, 7*S*, 10*S*-isomer, opposite of the data for **1** (Figure 4), indicating **2 **to be in agreement with 3*R*, 7*S* and 10*S*.

Compound **3** was isolated as a colorless oil. The ion peak was observed in ESI-MS at *m/z* 291 [M + Na]^+^, 559 [2M + Na]^+^. The ^1^H NMR spectrum indicated the presence of one methoxyl singlet (δ_H_ 3.75, s, H-15), two methyl singlets (δ_H_ 2.00, H-13, 1.72, H-14) and one terminal methyl triplet (δ_H_ 0.83, t, *J* = 7.0 Hz, H-12) (Table 2). The ^13^C NMR and HMQC spectra of **3** showed the presence of four methyls, five methylenes one methine, as well as five quaternary carbons, including two olefinic carbons (δ_C_ 168.7, 135.5), one oxygen-bearing quaternary carbon (δ_C_ 80.4), one ketone (δ_C_ 202.8) and one carbonyl (δ_C_ 175.2). The ^1^H and ^13^C NMR spectra of **3 **were almost the same as those of sinularone B [21]. The distinction was attributed to the presence of a methyl ester to replace an ethyl ester of the known analogue, as is evident from the molecular weight of **3**, to be 14 amu less than that of the latter, as well as the presence of a methoxyl group in its NMR spectra (Figure 2). 

Compound **4** was isolated as a colorless oil. The ion peak was observed in ESI-MS at *m/z* 253 [M − H]^−^. The ^1^H and ^13^C NMR spectra of **4 **were almost the same as those of **3**, except for the absence of a methoxyl group at C-15 (δ_C_ 52.4) in **4** (Table 2).

Compound **5** was isolated as a colorless oil. The ion peak was observed in ESI-MS at *m/z* 299 [M + H]^+^, 321 [M + Na]^+^. Comparison with **5 **showed almost the same NMR spectroscopic data as **3**, except for the presence of a methoxyl carbon at δ_C_ 54.7 and a oxygenated methine at δ_C _86.9, while a methylene signal appeared at δ_C_ 37.0 in **3** (Table 2). This implied that a methoxyl group is located at C-8 in **5 **instead of the methylene group in **3**. The assumption was confirmed by the correlations of CH_3_-15 to C-8 and H-8 to C-9 and C-3 in the HMBC experiment. 

Compound **6** was isolated as a colorless oil. The ion peak was observed in ESI-MS at *m/z* 283 [M − H]^−^. Close comparison of the ^13^C NMR spectrum of Compound **6 **to that of **5 **showed a general similarity, except for the absence of a methoxyl carbon at δ_C_ 54.7 at the C-16 position in **5** (Table 2). The measured CD curve of **3**–**6 **was very similar to the calculated ECD for 4*R*, 5*R*-isomer and opposite of the data for sinularone B [21], indicating **3**–**6 **to be in agreement with 4*R* and 5*R*.

Compound **7** was isolated as a colorless oil. The ion peak was observed in ESI-MS at *m/z*: 279 [M + Na]^+^, 535 [2M + Na]^+^. The ^1^H NMR spectrum suggested the presence of two methyl singlets (δ_H_ 1.85, s, H-10, 11) and two methoxyl singlets (δ_H_ 3.66, s, H-12, 3.08, s, H-13). The ^13^C NMR spectrum indicated the presence of four methyls, four methylenes and five quaternary carbons, including two carbonyl groups, two olefinic carbons and one oxygen-bearing quaternary carbon. Both the ^1^H and ^13^C NMR spectra of **7** showed a close similarity to 2,3-dimethyl butenolide [22,23], except for the absence of eight methylenes in the methoxycarbonyl side chain and the presence of an additional methoxy group. Comparison of the ^1^H and ^13^C NMR spectrum of **7** and **8** revealed that there were two fewer methylenes in the side chain in **8**. The negative specific rotation and the opposite Cotton effect in comparison with those of sinularone H indicated that C-4 had an *R* configuration [21,22].

By comparing the ^1^H, ^13^C-NMR and MS data with the literature values, the known Compounds **9** and **10** were identified as *ã*-methoxy-2,3-dimethyl-butenolide [24] and sinularone D [21], respectively.

**Table 2:** ^1^H and ^13^CNMR spectroscopic data for compounds **3**–**6** (500/125 MHz, in CDCl_3_, δ in ppm, *J* in Hz).

Position	3		4		5		6	
	^13^C	^1^H	^13^C	^1^H	^13^C	^1^H	^13^C	^1^H
1	175.2		174.3		175.5		174.4	
2	29.8	3.15 dd (4.5, 15.5)	31.7	2.98 m	29.6	3.05 dd (3.0, 18.0)	28.6	3.05 dd (4.5, 8.0)
		2.39 dd (7.0, 11.5)		2.62 d (15.5)		2.69 dd (6.5, 11.5 )		2.61 dd (4.5, 14.5)
3	55.0	2.85 dd (3.5, 8.5)	46.6	2.93 m	60.9	2.98 dd (3.0, 11.5)	60.3	2.94 dd (7.0,12.5)
4	202.8		204.1		203.1		204.4	
5	135.5		139.0		136.9		140.2	
6	168.7		167.1		165.9		165.7	
7	80.4		92.3		83.7		80.8	
8	37.0	1.76 td (19.0, 5.5)	34.5	1.97 td (14.0, 4.0)	86.9	3.29 dd (4.5, 8.0)	93.0	3.61 dd (2.5, 8.5)
		1.52 td (13.0, 3.5)		1.81 m				
9	25.1	0.75 m	23.3	0.88 m	31.3	1.64 m	30.6	1.68 m
		0.63 m		1.10 m		1.22 m		1.30 m
10	31.9	1.19 m	32.5	1.32 m	32.0	1.24 m	32.2	1.34 m
11	22.4	1.19 m	22.4	1.25 m	22.6	1.39 m	22.8	1.34 m
						1.07 m		1.51 m
12	13.9	0.83 t (7.0)	13.9	0.89 t (7.0)	13.9	0.83 t (7.0)	14.0	0.92 t (7.0 )
13	7.8	1.72 s	8.2	1.75 s	8.0	1.72 s	8.3	1.75 s
14	11.5	2.00 s	12.2	2.06 s	11.8	1.99 s	13.0	2.06 s
15	52.4	3.75 s			52.1	3.74 s	44.0	3.38 s
16					54.7	3.42 s		

Using HTS, all compounds were tested toward Forkhead box O 3α (Foxo3α), 3-hydroxy-3-methylglutaryl CoA reductase gene fluorescent protein (HMGCR-GFP), nuclear factor kappa B (NF-κB) luciferase, peroxisome proliferator-activated receptor-g co-activator 1α (PGC-1α), protein-tyrosine phosphatase 1B (PTP1B), mitochondrial membrane permeabilization (MMP) and adenosine monophosphate-activated protein kinase (AMPK) activity. Compounds **3**–**8** and **10** were evaluated for inhibition of NF-κB activation, and the inhibitory rates are listed in Table 3. At a concentration of 10 μg/mL, sinulolide E and sinularone D exhibited moderate effects with inhibitory rates of 38.12% and 43.00%, respectively. However, all compounds were inactive against other biological targets.

**Table 3:** Inhibitory rates of NF-κB activation of Compounds **3**–**8** and **10**.

Concentration	IR (%)						
	3	4	5	6	7	8	10
10 μg/mL	27.85	28.75	38.12	28.24	27.08	25.28	43.00

## 3. Experimental Section 

### 3.1. General Experimental Procedures

The NMR spectra were recorded on a Bruker AC 500NMR spectrometer (Bruker BioSpin, Fällanden, Switzerland) with tetramethylsilane (TMS) as an internal standard. ESI-MS data were measured on a Bruker amaZon SL spectrometer (Bruker, Fällanden, Switzerland). HR-ESI-MS data were measured on a Bruker micro TOF-QII mass spectrometer (Bruker, Fällanden, Switzerland). CD spectra were measured with a Chirascan circular dichroism spectrometer (Applied Photophysics Ltd., Leatherhead, UK). Optical rotation values were measured with an Anton Paar MCP500 polarimeter (Anton Paar, Graz, Austria). YMC gel (ODS-A, 12 nm, S-50 µm, YMC, Kyoto, Japan) was used for column chromatography. The SiO_2_ GF_254_ used for TLC was supplied by the Qingdao Marine Chemical Factory, Qingdao, China. Sephadex LH-20 gel (GE Healthcare, Uppsala, Sweden) was used. HPLC was carried out on a Hitachi L-2400 (Hitachi, Tokyo, Japan) with a YMC ODS column. Spots were detected on TLC under UV light or by heating after spraying with 5% H_2_SO_4_ in EtOH (v/v).

### 3.2. Animal Material

The soft coral *Sinularia* sp. was collected from Dongluo Island, Hainan province of China, in July, 2009 (7–10-m depth) and identified by Professor Hui Huang, South China Sea Institute of Oceanology, Chinese Academy of Sciences. A voucher specimen (No. 0907010) was deposited in the CAS Key Laboratory of Tropical Marine Bio-resources and Ecology, South China Sea Institute of Oceanology, Chinese Academy of Sciences, Guangzhou, China.

### 3.3. Extraction and Isolation

The fresh soft coral (wet, 6 kg) was extracted three times with 95% EtOH (20 L). The extract was concentrated under reduced pressure and partitioned between H_2_O (4 L) and CHCl_3_ (4 L); the CHCl_3_ layer (120 g) was further partitioned between 85% EtOH (4 L) and petroleum ether (PE; 4 L) to yield 85% EtOH (34 g) and PE (75.6 g) fractions. The 85% EtOH fraction was separated by silica gel column using CHCl_3_/MeOH to yield 11 portions (Fr. s1–s11). Fr. s3 was purified by silica gel column to yield 12 portions, and Portion 10 was further purified with semi-preparative HPLC, eluting with MeOH/H_2_O = 65:35 at a flow rate of 2 mL/min, to afford **1 **(4.5 mg) and **2 **(2.4 mg). Fr. s5 was purified by Sephadex LH-20 using CHCl_3_/MeOH = 1:1 to yield 3 portions, and Portion 3 was further purified with semi-preparative HPLC, eluting with MeOH/H_2_O = 57:43 at a flow rate of 2 mL/min, to afford **7 **(3.0 mg), **8** (4.1 mg) and **9** (10.0 mg). Fr. s6 was further purified with semi-preparative HPLC, eluting with MeOH/H_2_O = 60:40 at a flow rate of 2 mL/min, to afford **3 **(2.4 mg), **4 **(2.8 mg) and **10** (6.7 mg). Fr. s7 was purified by Sephadex LH-20 to yield three portions, and Portion 3 was further purified with semi-preparative HPLC, eluting with MeOH/H_2_O = 60:40 at a flow rate of 2 mL/min, to afford **5 **(3.5 mg) and **6** (4.2 mg).

Sinulolide A (**1**): colorless oil; ${\text{[α]}}_{\text{D}}^{\text{25}}$ = −172.5 (*c* = 0.35, MeOH); CD (MeOH; *c* 0.2): Δε287 −9.72, Δε*2*46 +12.72, Δε226 −0.76; ^1^H and ^13^C NMR data: see Table 1; HRESIMS *m*/*z* 367.2117 [M + H]^+^ (calcd. for C_20_H_3__1_O_6_, 367.2115), 389.1942 [M + Na]^+^ (calcd. for C_20_H_30_O_6_Na, 389.1935). 

Sinulolide B (**2**): colorless oil; ${\text{[α]}}_{\text{D}}^{\text{25}}$ = +103.2 (*c* = 0.07, MeOH); CD (MeOH; *c* 0.2): Δε287 +3.94, Δε*2*46 −4.66, Δε226 +0.51; ^1^H and ^13^C NMR data: see Table 1; ESI-MS *m/z* 367 [M + H]^+^, 389 [M + Na]^+^, 755 [2M + Na]^+^.

Sinulolide C (**3**): colorless oil; ${\text{[α]}}_{\text{D}}^{\text{25}}$ = −3.2 (*c* = 0.01, MeOH); CD (MeOH; *c* 0.2): Δε210 −0.23; ^1^H and ^13^C NMR data: see Table 2; ESI-MS *m/z* 291 [M + Na]^+^, 559 [2M + Na]^+^.

Sinulolide D (**4**): colorless oil; ${\text{[α]}}_{\text{D}}^{\text{25}}$ = −2.5 (*c* = 0.01, MeOH); CD (MeOH; *c* 0.2): Δε207 –0.48, Δ*ε*238 −0.46; ^1^H and ^13^C NMR data: see Table 2; ESI-MS *m/z* 253 [M – H]^−^.

Sinulolide E (**5**): colorless oil; ${\text{[α]}}_{\text{D}}^{\text{25}}$ = −3.8 (*c* = 0.01, MeOH); CD (MeOH; *c* 0.2): Δε218 –0.52; ^1^H and ^13^C NMR data: see Table 2; ESI-MS *m/z* 299 [M + H]^+^, 321 [M + Na]^+^.

Sinulolide F (**6**): colorless oil; ${\text{[α]}}_{\text{D}}^{\text{25}}$ = −6.6 (*c* = 0.01, MeOH); CD (MeOH; *c* 0.2): Δε206 −0.48; ^1^H and ^13^C NMR data: see Table 2; ESI-MS *m/z* 283 [M − H]^ −^.

Sinulolide G (**7**): colorless oil; ${\text{[α]}}_{\text{D}}^{\text{25}}$ = −4.4 (*c* = 0.05, MeOH); CD (MeOH; *c* 0.2): Δε229 −0.4; ^1^H NMR (500 MHz, CDCl_3_) δ: 3.66 (3H, s), 3.08 (3H, s), 2.29 (2H, m), 1.99 (1H, m), 1.85 (6H, s), 1.70 (1H, m), 1.61 (2H, m), 1.23 (2H, m); ^13^C NMR (125 MHz, CDCl_3_) δ: 173.4 (C, C-9), 171.9 (C, C-1), 155.6 (C, C-3), 127.4 (C, C-2), 109.5 (C, C-4), 51.6 (CH_3_, C-10), 50.1 (CH_3_, C-11), 35.3 (CH_2_, C-5), 33.8 (CH_2_, C-8), 24.8 (CH_2_, C-6), 22.3 (CH_2_, C-7), 10.7 (CH_3_, C-13), 8.5 (CH_3_, C-12); ESI-MS *m/z*: 279 [M + Na]^+^, 535 [2M + Na]^+^.

Sinulolide H (**8**): colorless oil; ${\text{[α]}}_{\text{D}}^{\text{25}}$ = −3.2 (*c* = 0.03, MeOH); CD (MeOH; *c* 0.2): Δε229 −0.4; ^1^H NMR (500 MHz, CDCl_3_) δ: 3.66 (3H, s), 3.07 (3H, s), 2.52 (1H, m), 2.39 (1H, m), 2.30 (1H, m), 1.96 (1H, m), 1.88 (3H, s), 1.85 (3H, s); ^13^C NMR (125 MHz, CDCl_3_) δ: 173.2 (C, C-7), 171.2 (C, C-1), 155.7 (C, C-3), 127.7 (C, C-2), 108.9 (C, C-4), 51.8 (CH_3_, C-8), 50.2 (CH_3_, C-9), 31.2 (CH_2_, C-5), 27.8 (CH_2_, C-6), 10.7 (CH_3_, C-11), 8.5 (CH_3_, C-10); ESI-MS at *m/z*: 251 [M + Na]^+^, 479 [2M + Na]^+^.

### 3.4. Assays for Bioactivities

Bioactivity assays were performed by the National Center for Drug Screening, the State Key Laboratory of Drug Research, Shanghai Institute of Materia Medica, Chinese Academy of Sciences, using HTS [25]. Previously reported procedures were followed for assaying the bioactivity against Foxo3α [26], HMGCR-GFP [27], NF-κB luciferase [28], PGC-1α [29], PTP1B [30], MMP [31] and AMPK [32].

### 3.5. Computational Calculation

The computational ECD, specific rotation and ^13^C NMR calculations were performed by the B3LYP functional and a generic basis set, employing the 6-311+G (*d*,*p*) basis set [21,33]. This generic basis set has been shown to be effective, both efficient and reliable, in predicting structural and reactivity properties for homogeneous systems. Molecular Merck force field (MMFF) and density functional theory/time dependent density functional theory (DFT/TDDFT) calculations were performed with Spartan’14 software package (Wavefunction Inc., Irvine, CA, USA) and the Gaussian 09 program package, respectively, using default grids and convergence criteria.

## 4. Conclusions 

Our study revealed the chemical constituents of soft coral *Sinularia* sp., which is rich in the South China Sea. Ten compounds were isolated and purified, including seven cyclopentenone derivatives and three butenolide derivatives. Using HTS, their bioactivities toward several targets, such as Foxo3α, HMGCR-GFP, NF-κB-luciferase, PGC-1a, PTP1B, MMP and AMPK, were evaluated. Compounds **5** and **10** exhibited moderate effects for the inhibition of NF-κB activation.

## Supplementary Files

Supplementary File 1
