# Supplementary material for: Sinulolides A–H, New Cyclopentenone and Butenolide Derivatives from Soft Coral Sinularia sp
Source: Mar Drugs. 2014 Oct 23;12(10):5316–27. doi: 10.3390/md12105316 (PMC4210901; doi:10.3390/md12105316)
Supplement: Supplementary File 1 [file marinedrugs-12-05316-s001.pdf]

Supplementary Information

Figure S1. ^{1}H NMR Spectrum (500 MHz) of compound **1** in $CDCl_3$.

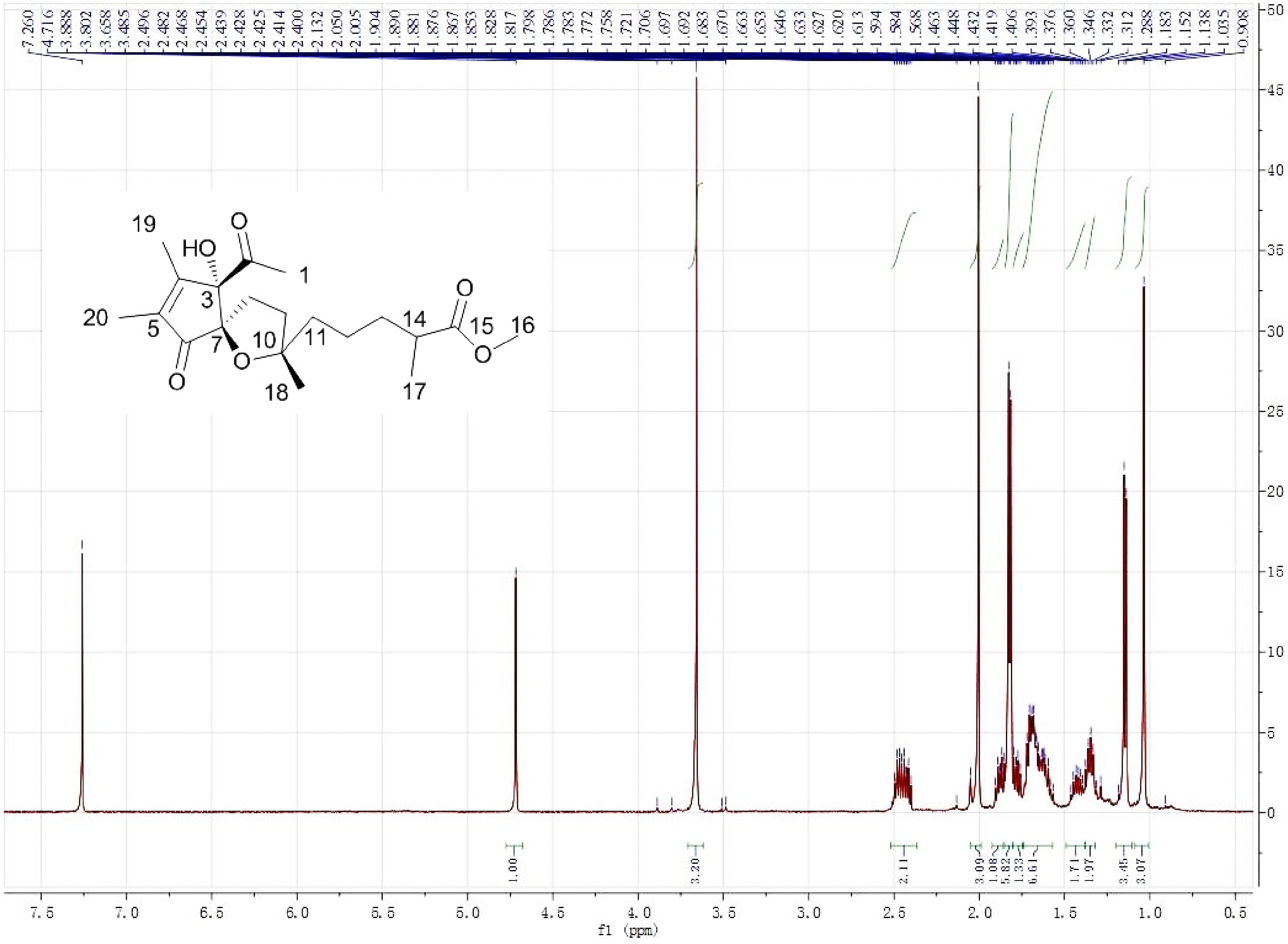

Figure S2. ^{13}C NMR Spectrum (125 MHz) of compound **1** in $CDCl_3$.

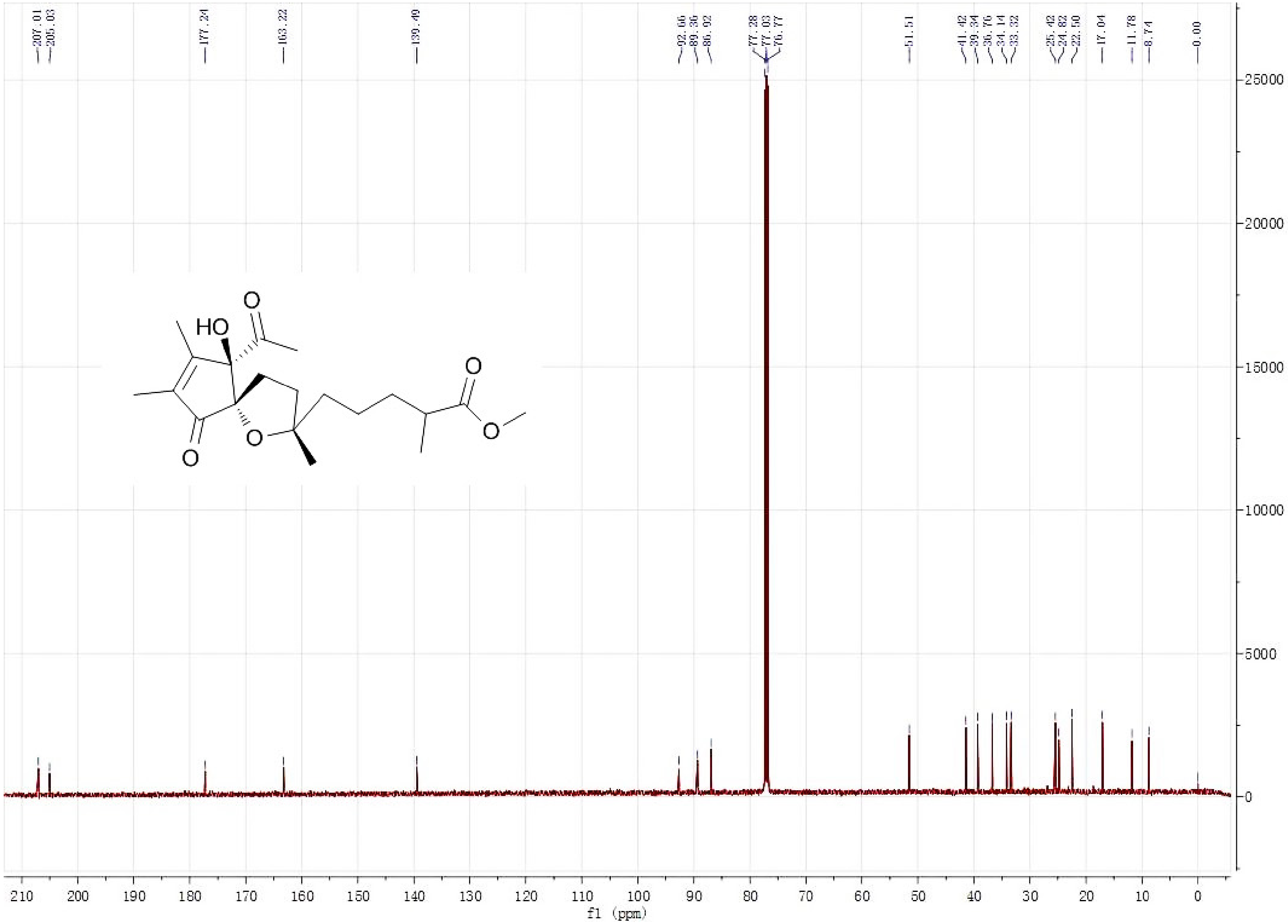

Figure S3.HSQC Spectrum of compound**1** in $CDCl_3$.

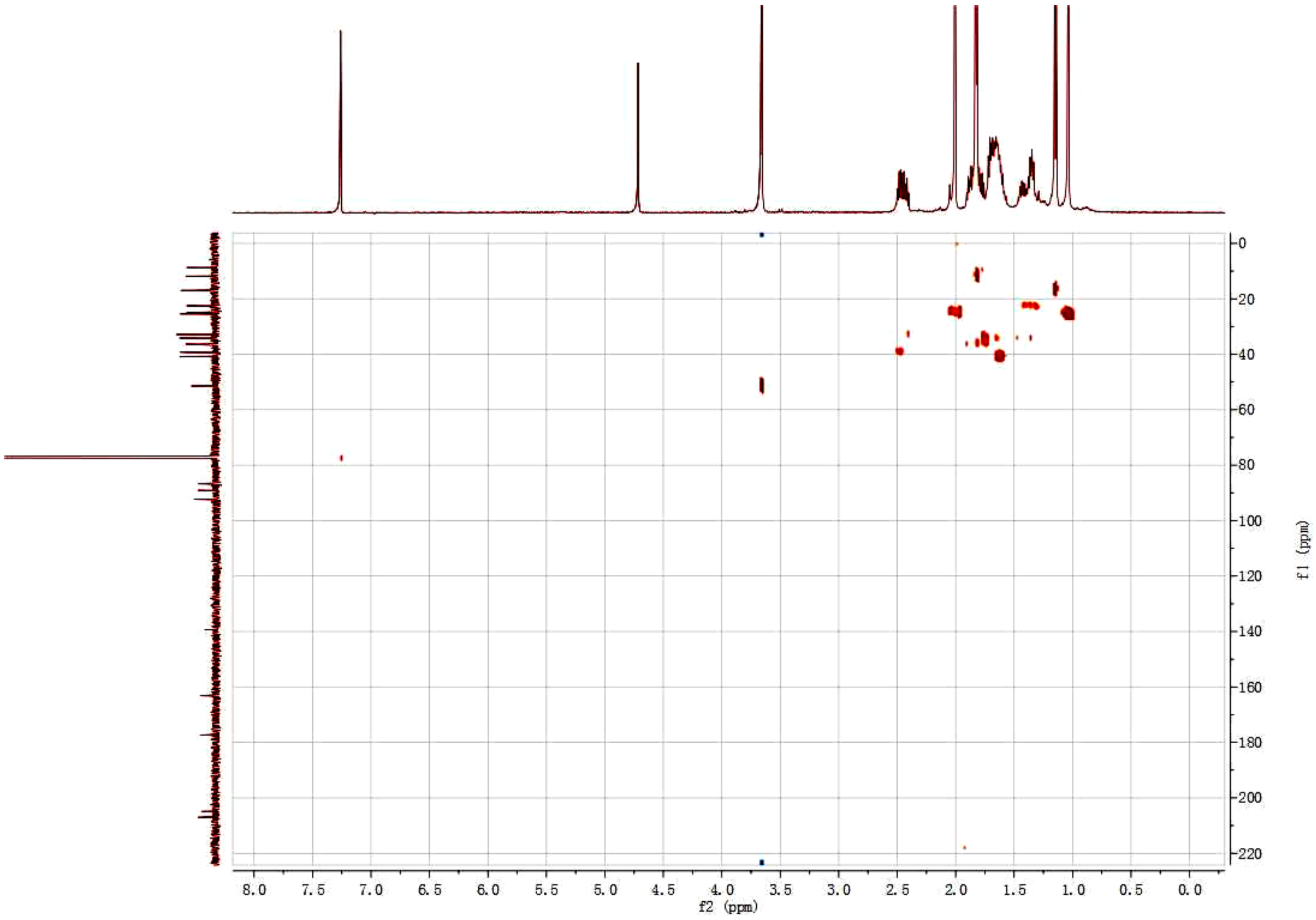

Figure S4. HMBC Spectrum of compound **1** in $CDCl_3$.

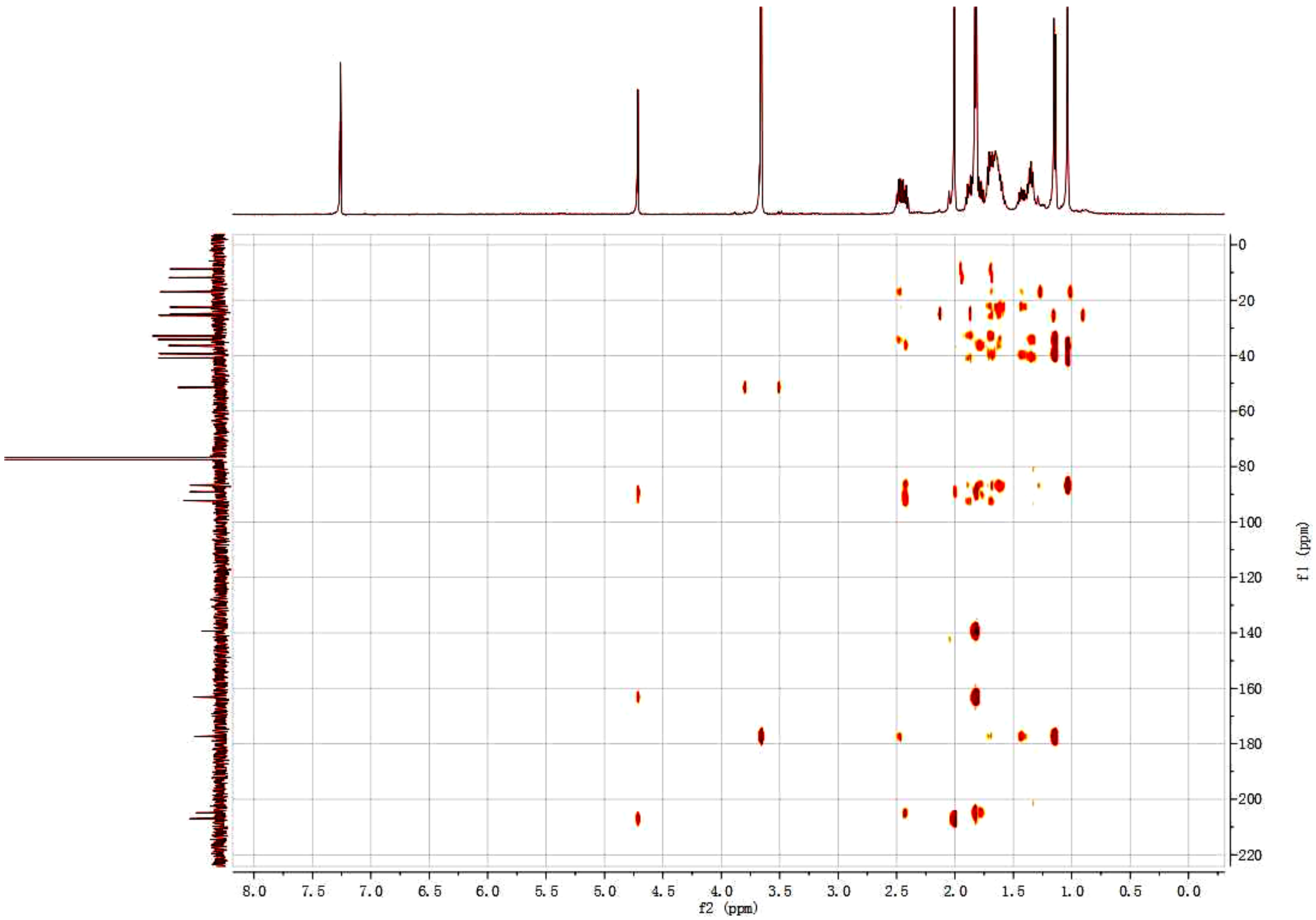

Figure S5.NOESY Spectrum of compound **1** in $CDCl_3$.

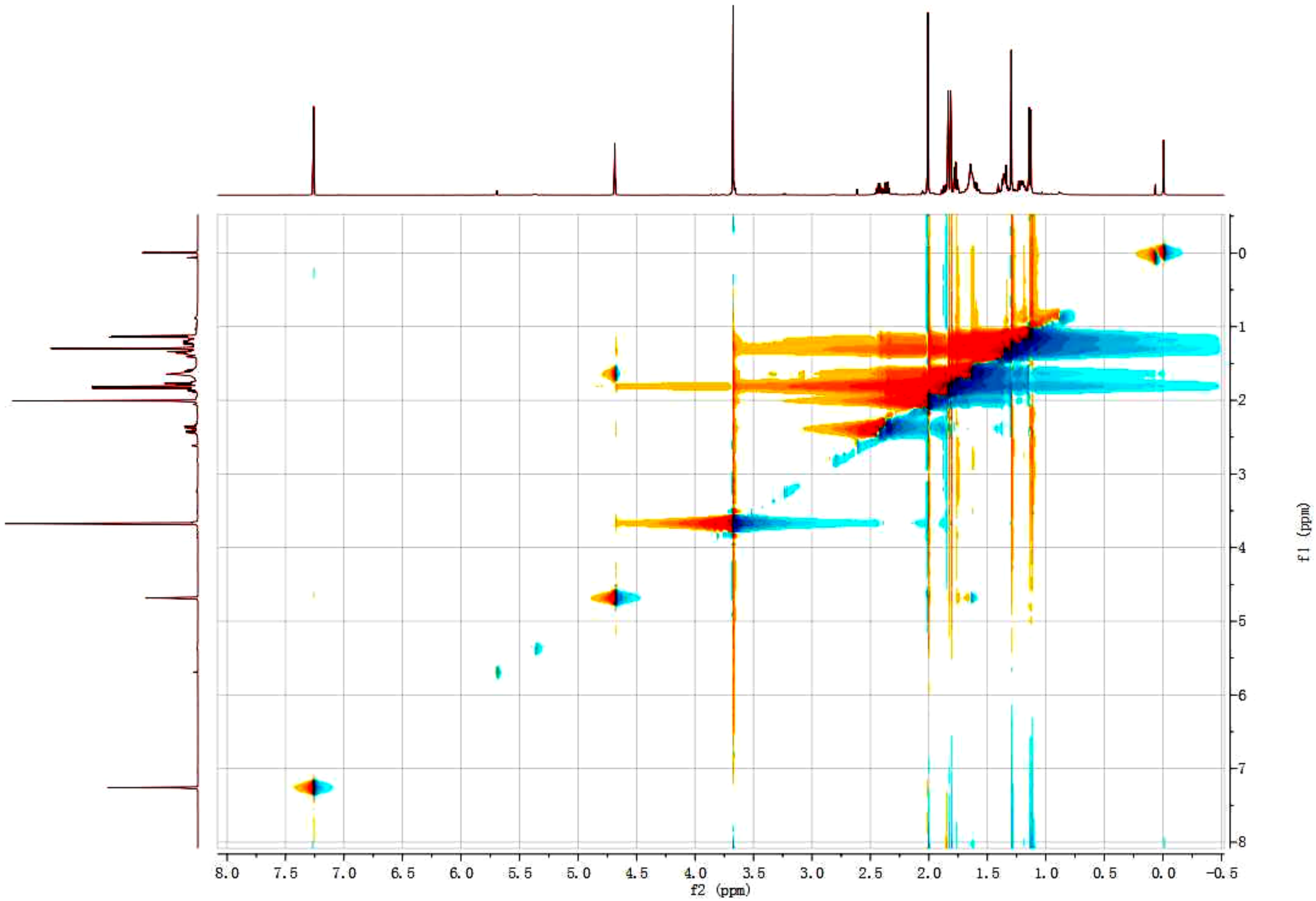

Figure S6. HRESIMS spectrum of compound **1**.

Mass Spectrum SmartFormula Report

Analysis Info

Analysis Name	D:\Data\MS\data\201403\yangbin-sa5104-2_pos.d	Acquisition Date	3/27/2014 9:35:42 AM
Method	POS_100-2000_Dirrect Infusion.m	Operator	SCSIO
Sample Name	SCSIO	Instrument / Ser#	maXis 29
Comment			

Acquisition Parameter

Source Type	ESI	Ion Polarity	Positive	Set Nebulizer	0.4 Bar
Focus	Not active	Set Capillary	4500 V	Set Dry Heater	180 °C
Scan Begin	100 m/z	Set End Plate Offset	-500 V	Set Dry Gas	4.0 l/min
Scan End	2000 m/z	Set Collision Cell RF	2000.0 Vpp	Set Divert Valve	Waste

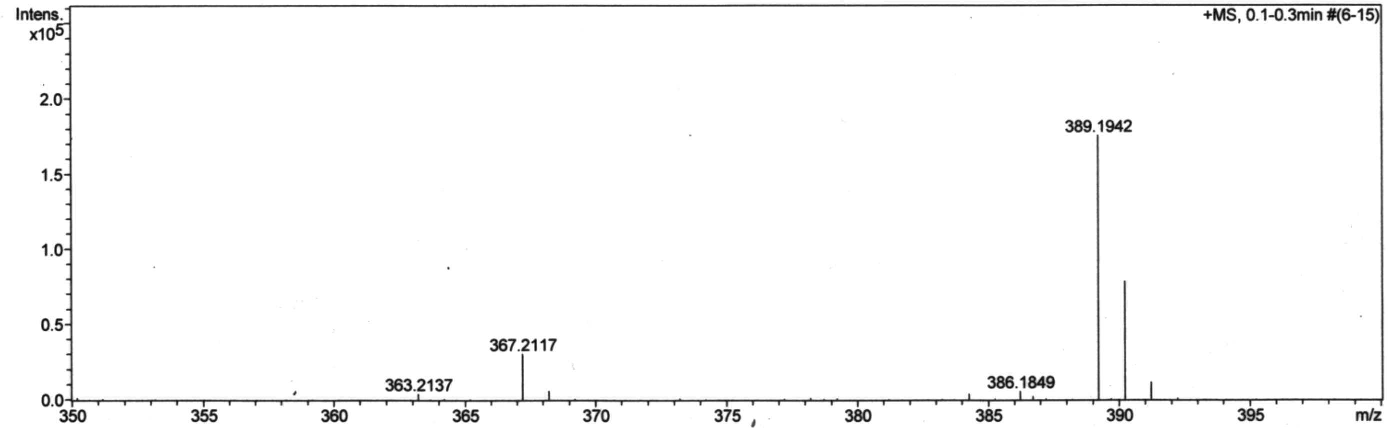

Meas. m/z	#	Formula	Score	m/z	err [mDa]	err [ppm]	mSigma	rdb	e⁻ Conf	N-Rule
367.2117	1	C 20 H 31 O 6	100.00	367.2115	-0.2	-0.5	15.7	5.5	even	ok
389.1942	1	C 20 H 30 Na O 6	100.00	389.1935	-0.7	-1.9	134.5	5.5	even	ok

Bruker Compass DataAnalysis 4.0 printed: 3/27/2014 9:39:30 AM Page 1 of 1

Figure S7. ^{1}H NMR Spectrum (500 MHz) of compound **2** in $CDCl_3$.

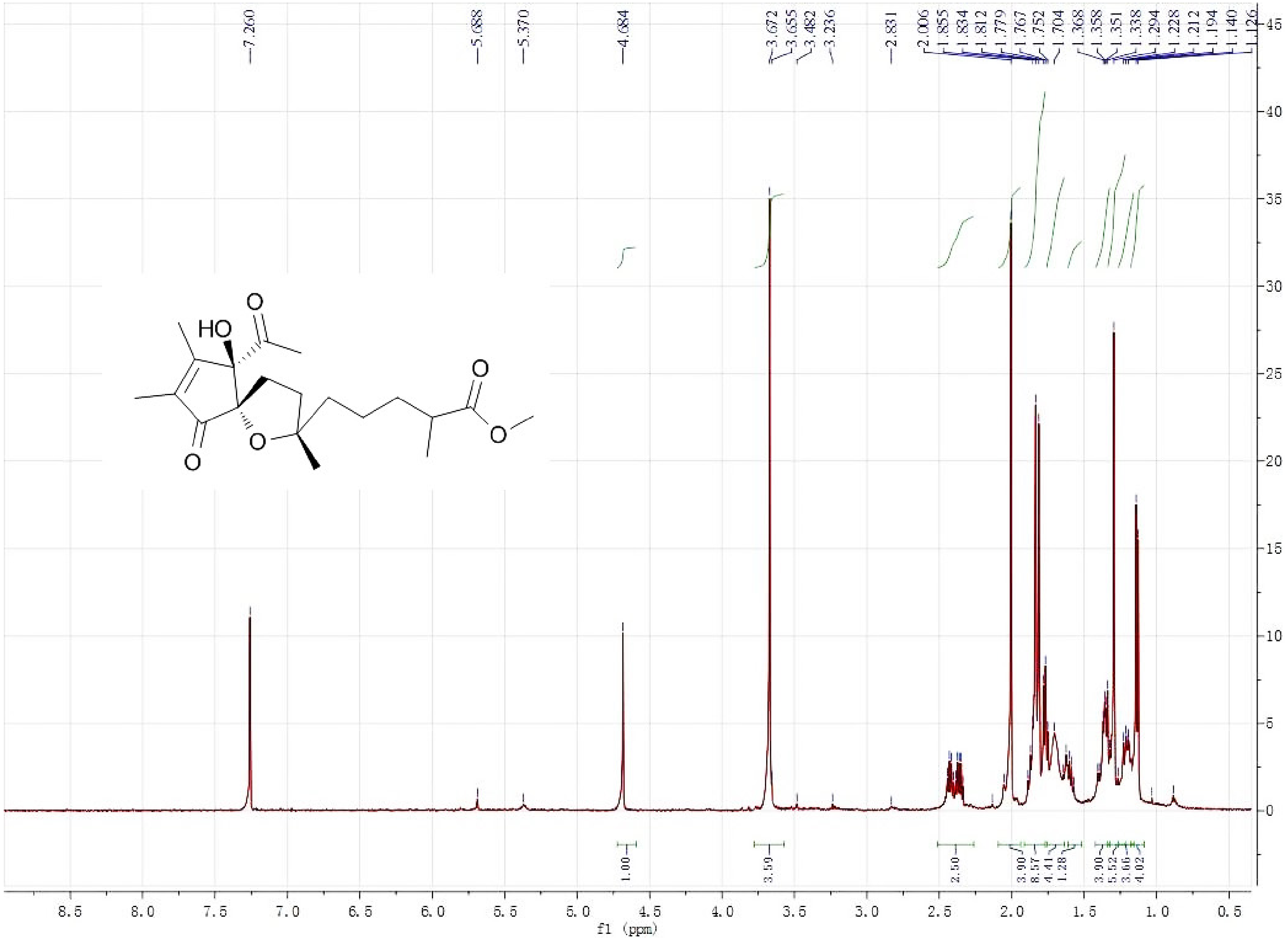

Figure S8.^{13}C NMR Spectrum (125 MHz) of compound **2** in $CDCl_3$.

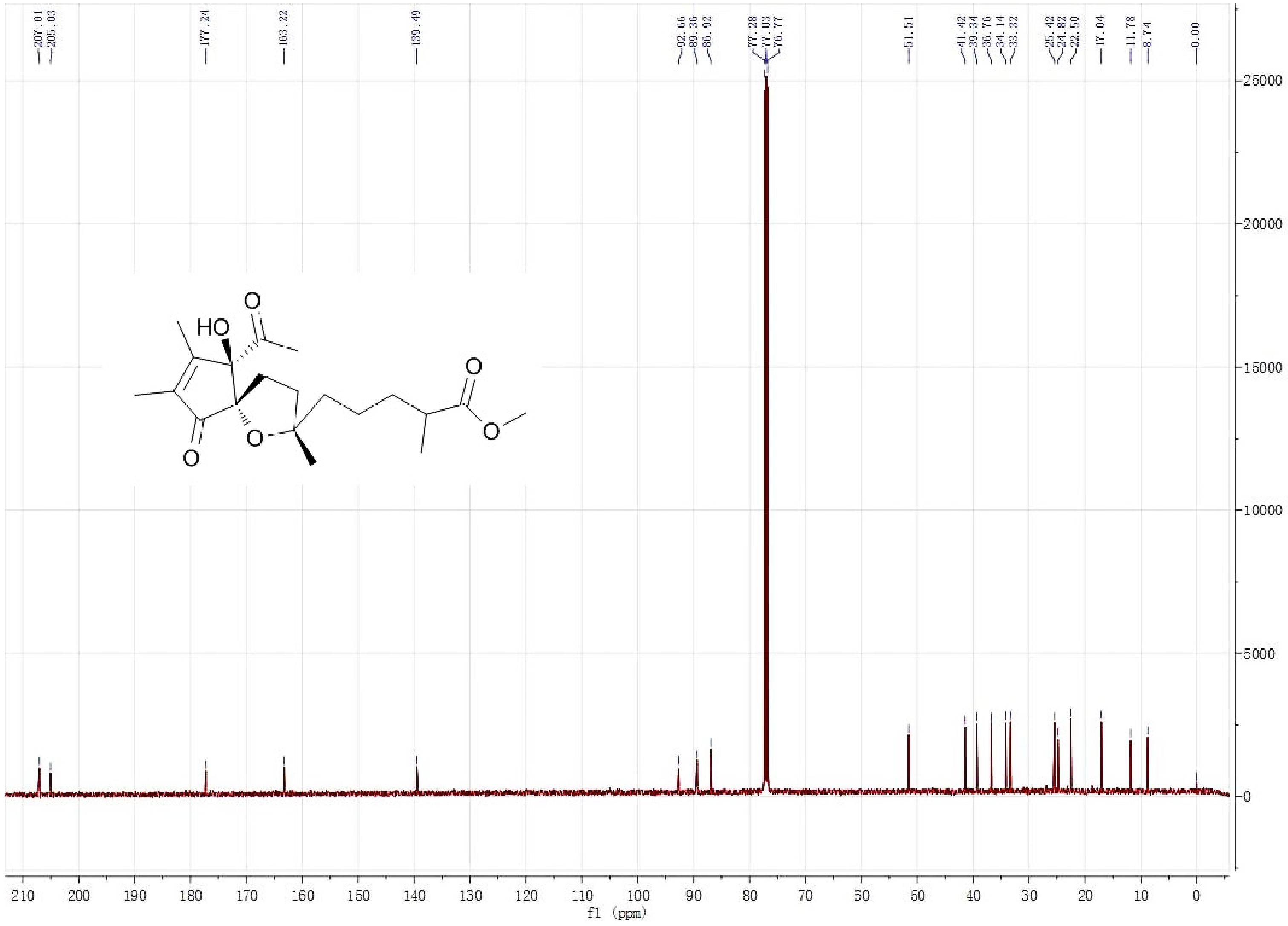

Figure S9. HSQC Spectrum of compound **2** in $CDCl_3$.

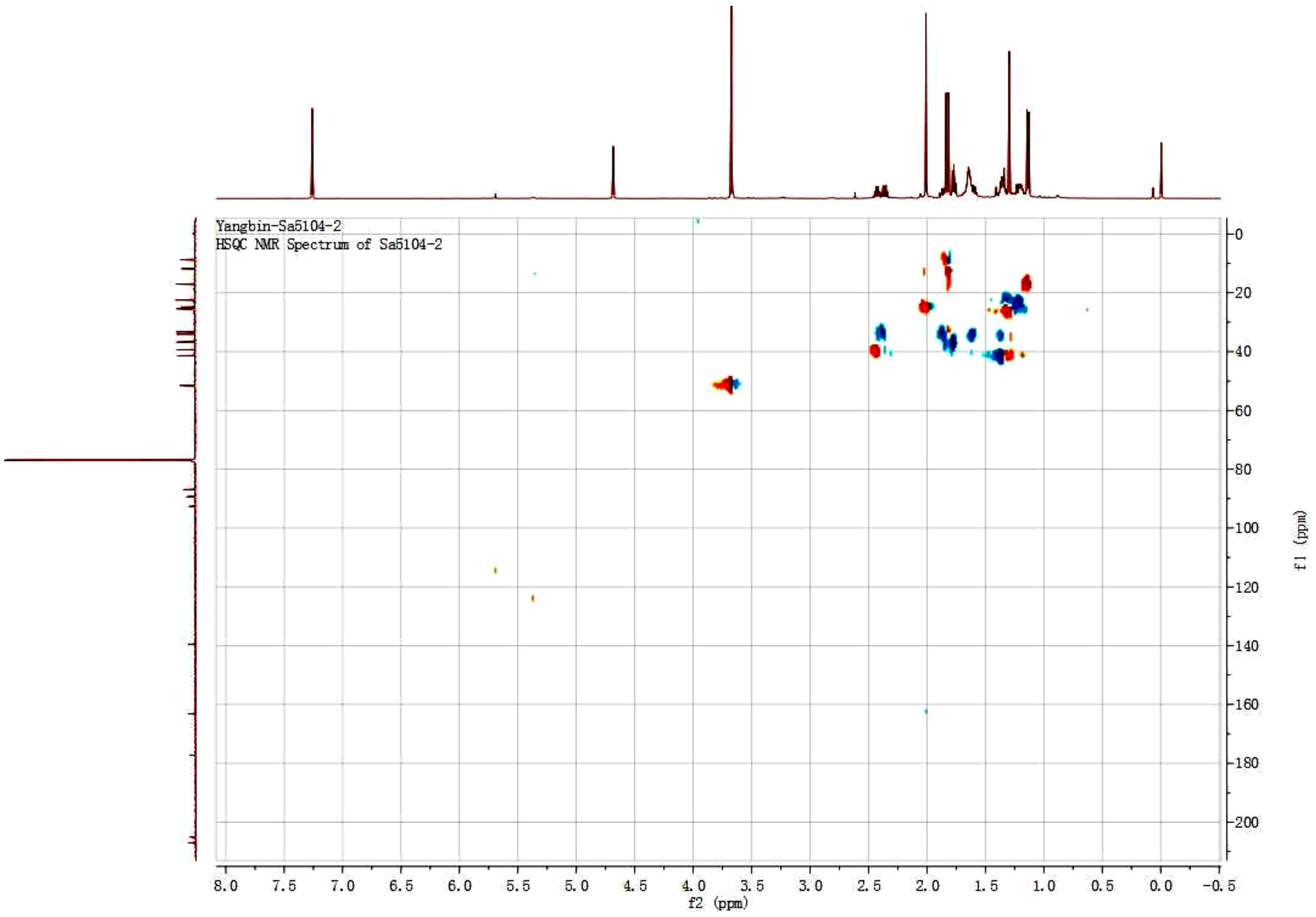

Figure S10. HMBC Spectrum of compound **2** in CDCl3.

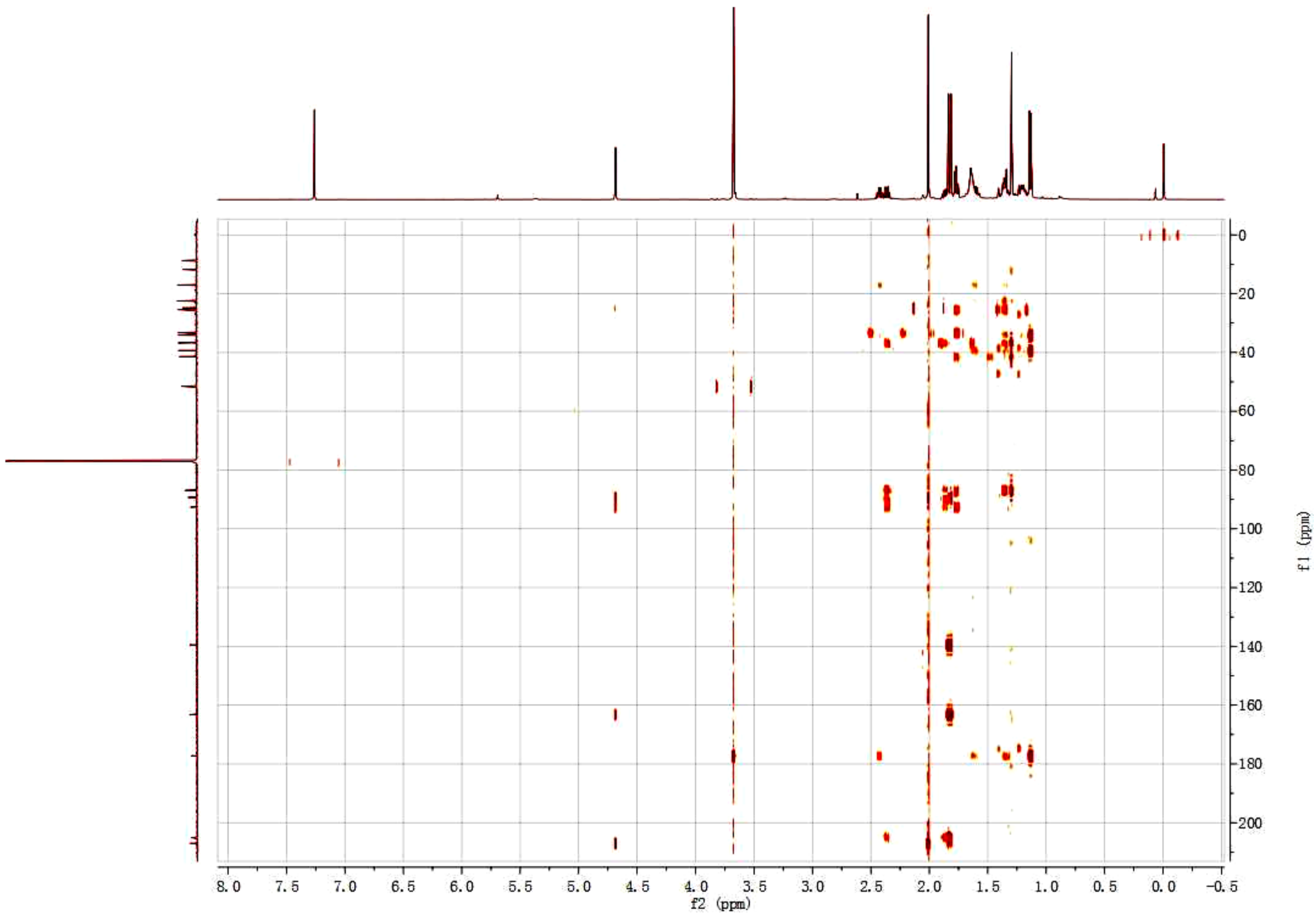

Figure S11. ESIMS Spectrum of compound **2**.

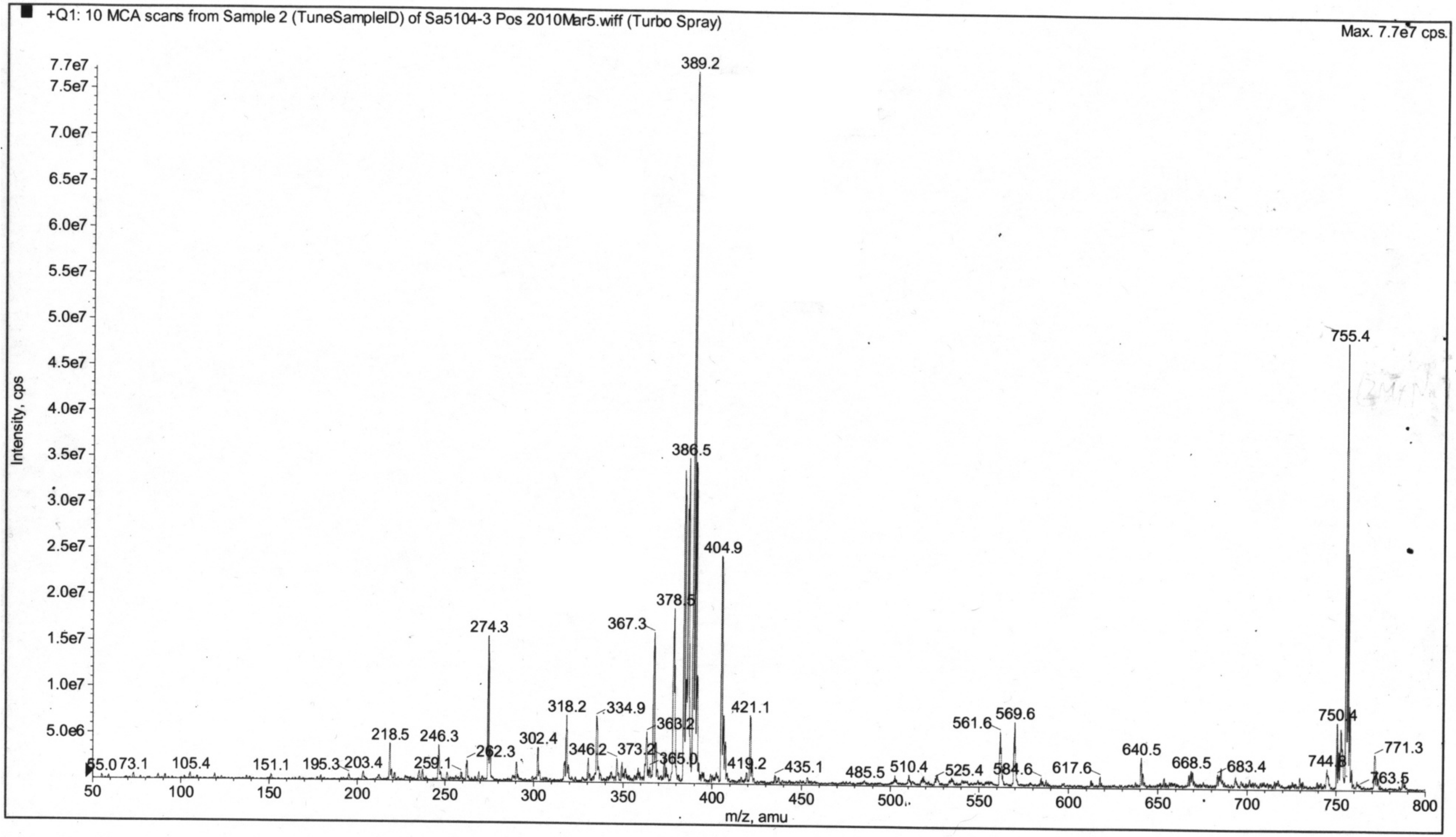

Figure S12. CD Spectrum of compound **1** and **2**.

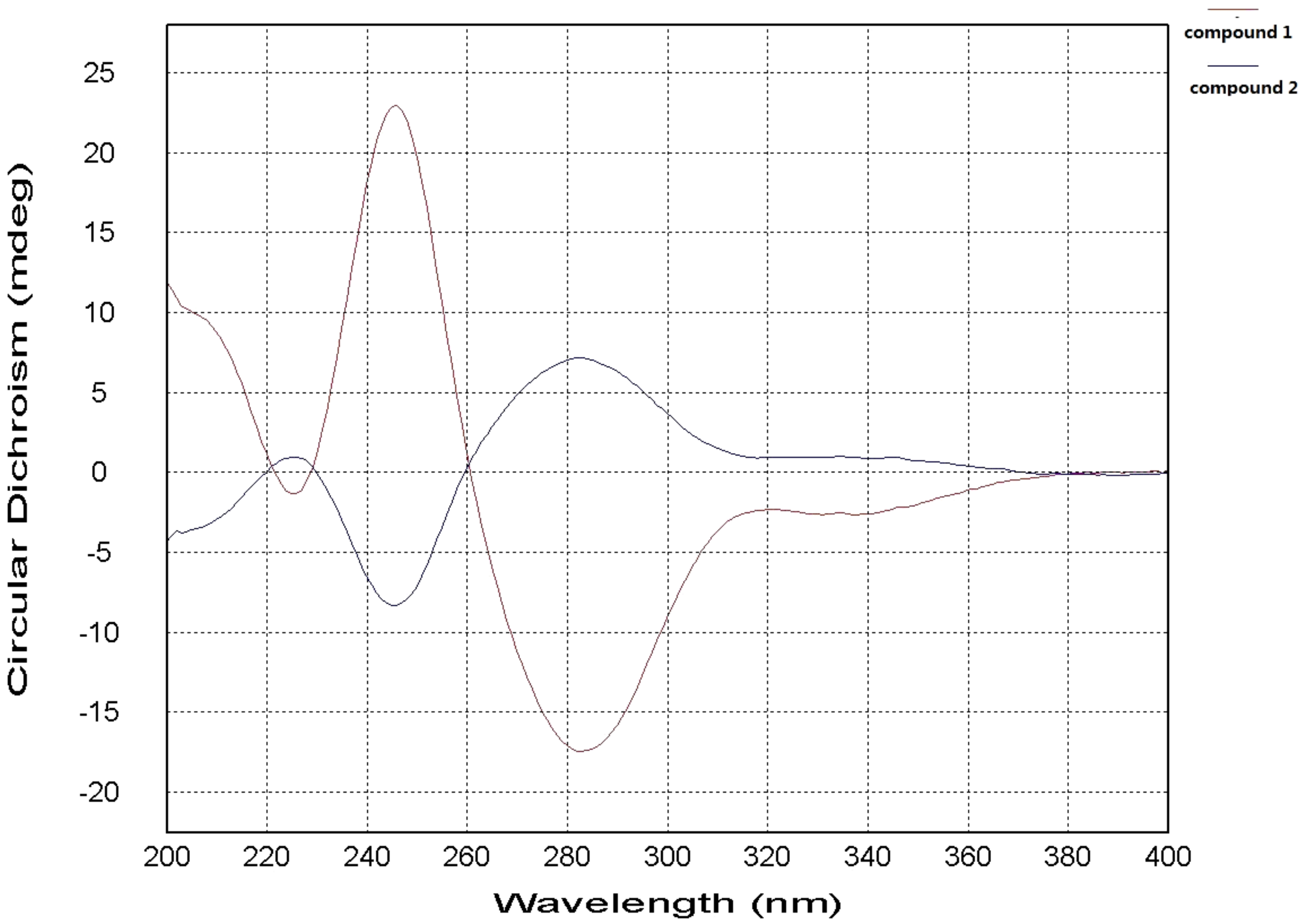

Table S1. Relative and free energies [a] and equilibrium populations [b] of low-energy conformers of **1** and **2**in MeOH.

**Conformer**	**Δ*E***	**Δ*G***	**P (%)**
Compound **1** (3*S*,7*R*,10*S*)			
**1a**	0.0	0.0	32.8
**1b**	0.017	0.170	24.5
**1c**	0.021	0.332	18.6
**1d**	0.170	0.397	16.6
**1e**	1.046	0.966	6.2
**1f** [c]	1.064	1.908	1.2
Compound **2** (3*S*,7*R*,10*R*)			
**2a**	0.00	0.00	32.6
**2b**	0.118	0.165	24.6
**2c**	0.411	0.196	23.3
**2d**	0.573	0.439	15.3
**2e**	0.938	1.192	4.2

[a] B3LYP/6-311+G(d,p), in kcal/mol; [b] From ΔG values at 298.15 K;[c] Conformer not used for ECD/TDDFT calculations.

Figure S13. Conformations of low-energy conformers of **1** and **2**.

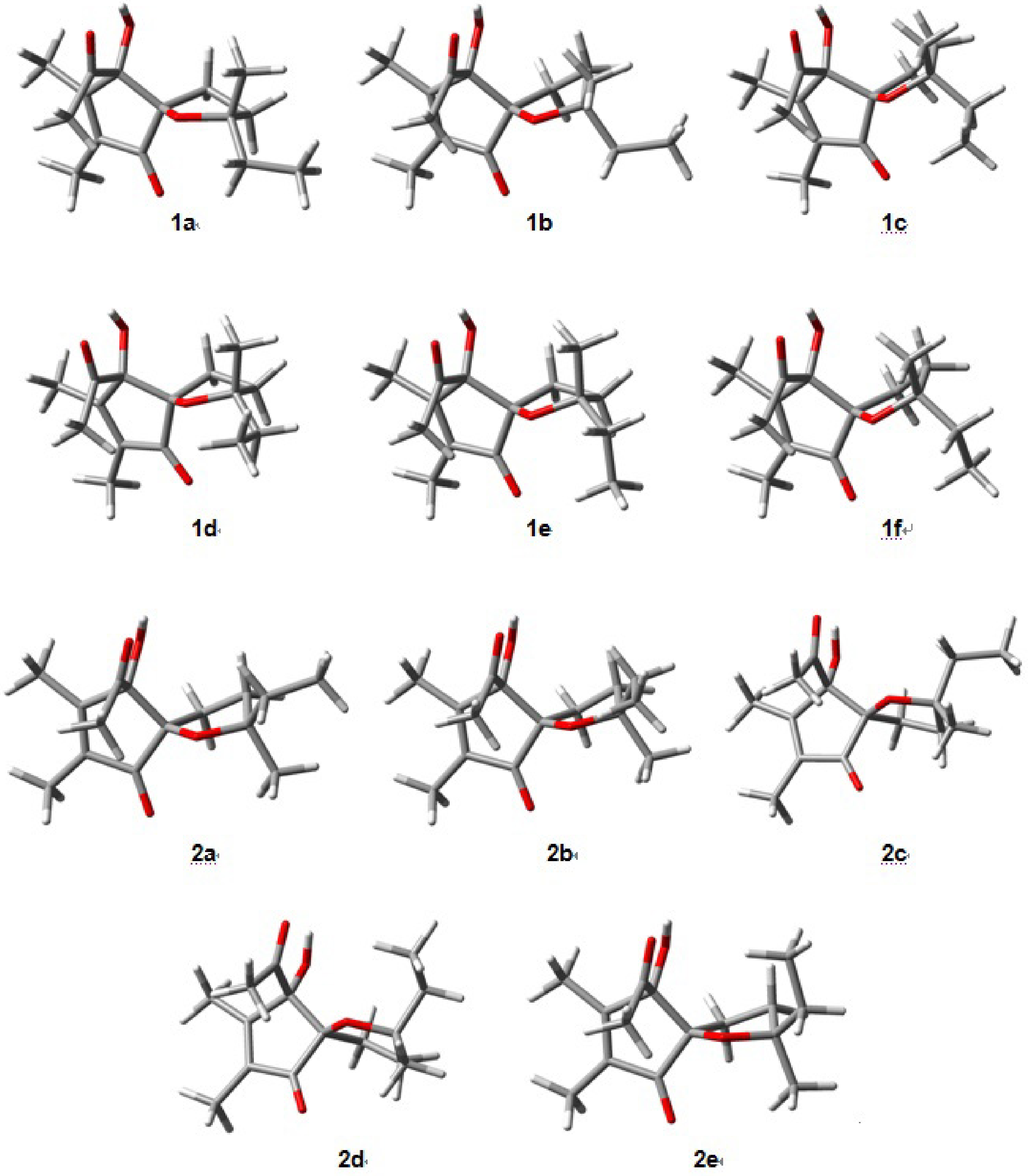

Figure S14. Calculated ECD Spectrum for the low-energy conformers of **1**. Vertical bars represent rotational strengths *R*. σ = 0.30 eV, shift = ±0 nm.

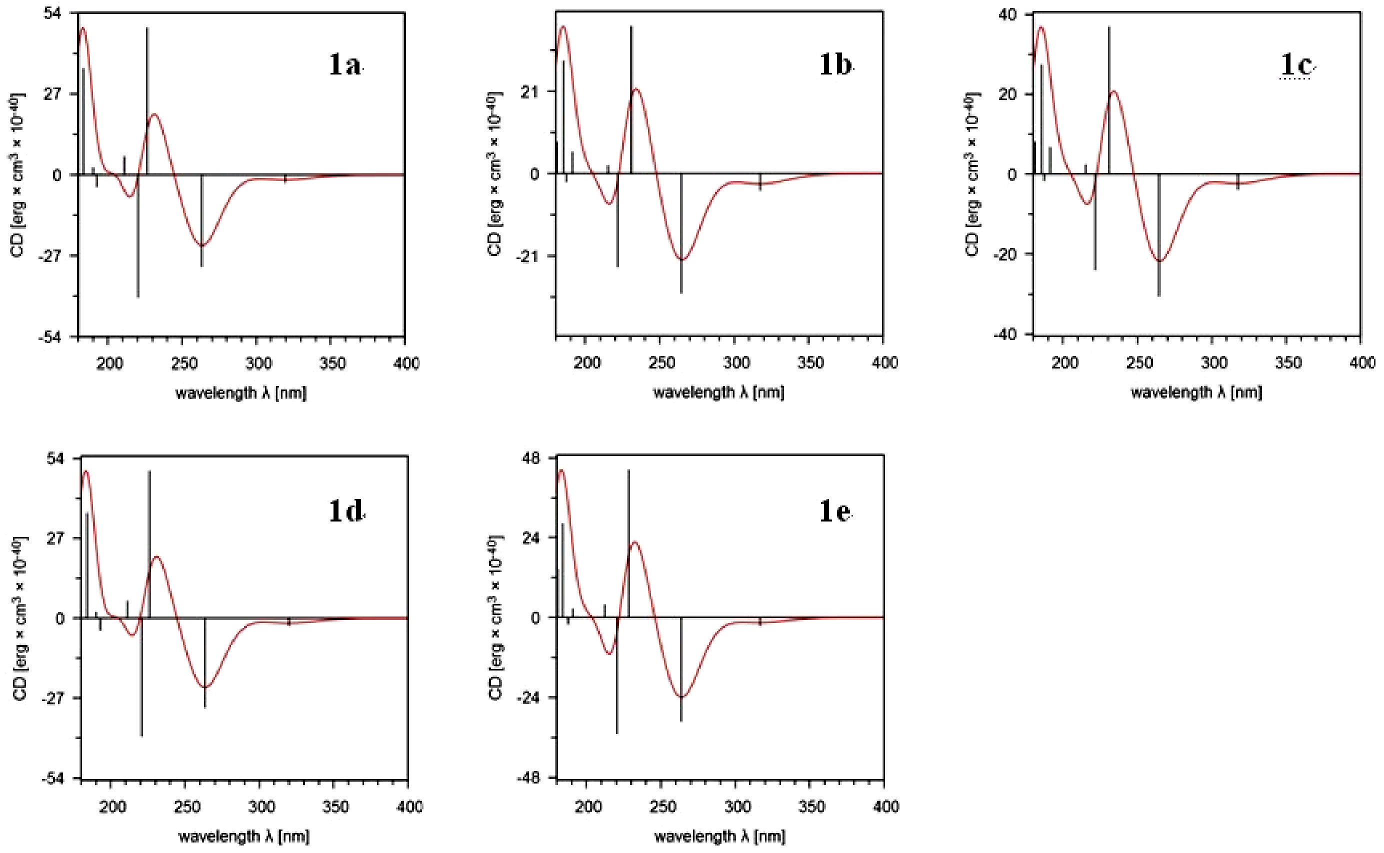

Figure S15. Calculated ECD Spectrum for the low-energy conformers of **2**. Vertical bars represent rotational strengths *R*. σ = 0.30 eV, shift = ±0 nm.

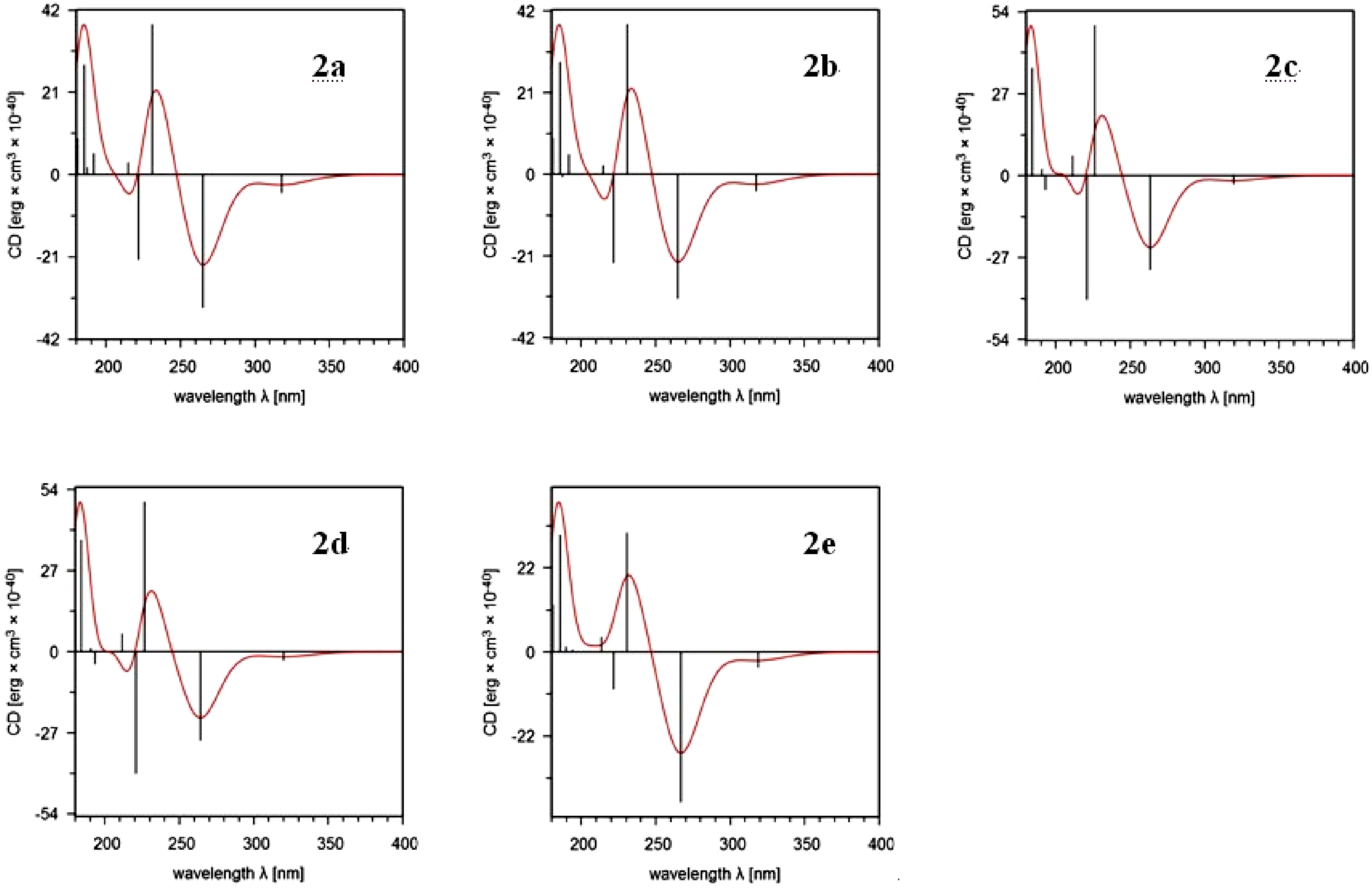

Figure S16.^{1}H NMR Spectrum (500 MHz) of compound **3** in $CDCl_3$.

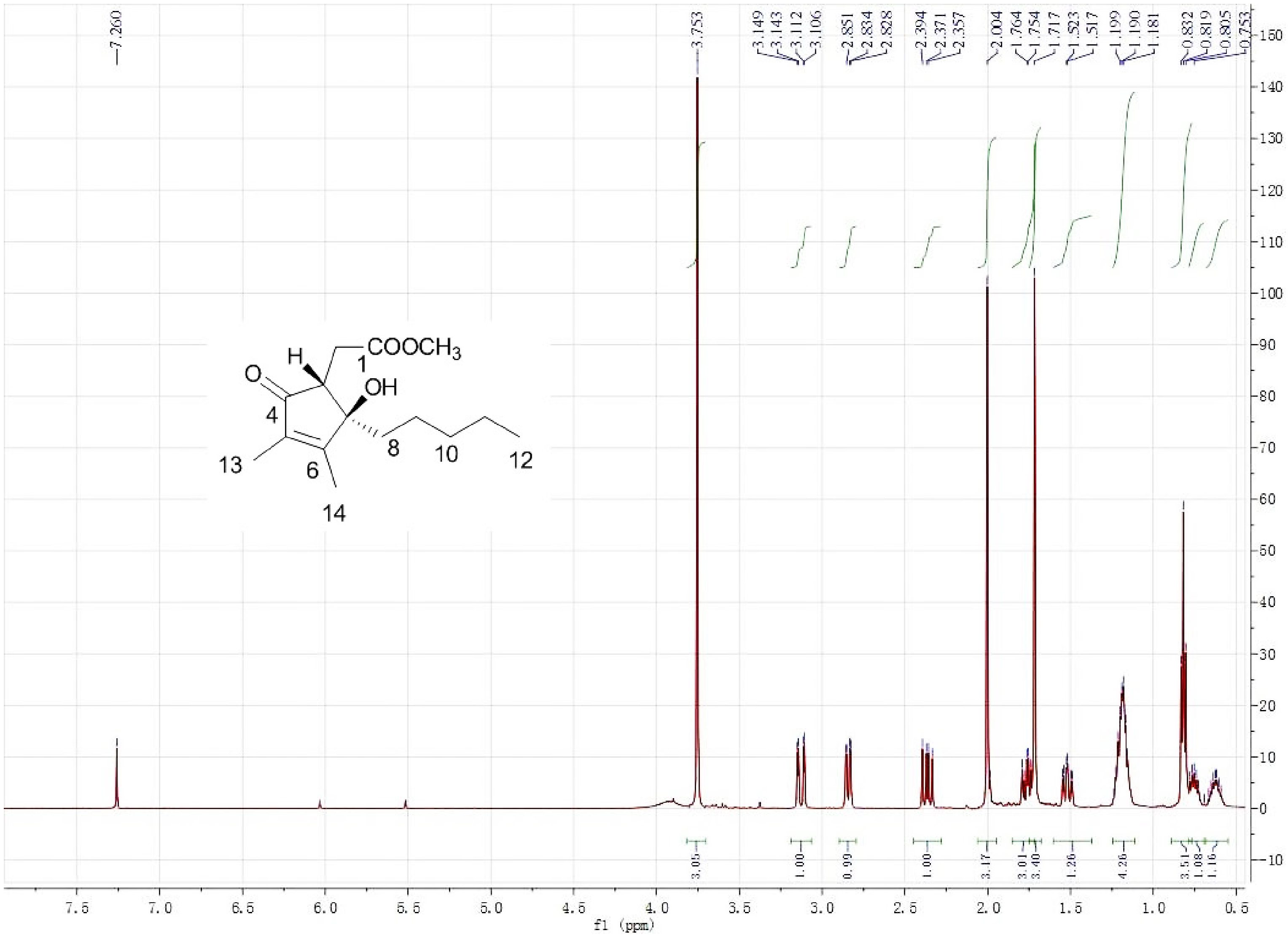

Figure S17. ^{13}C NMR Spectrum (125 MHz) of compound **3** in $CDCl_3$.

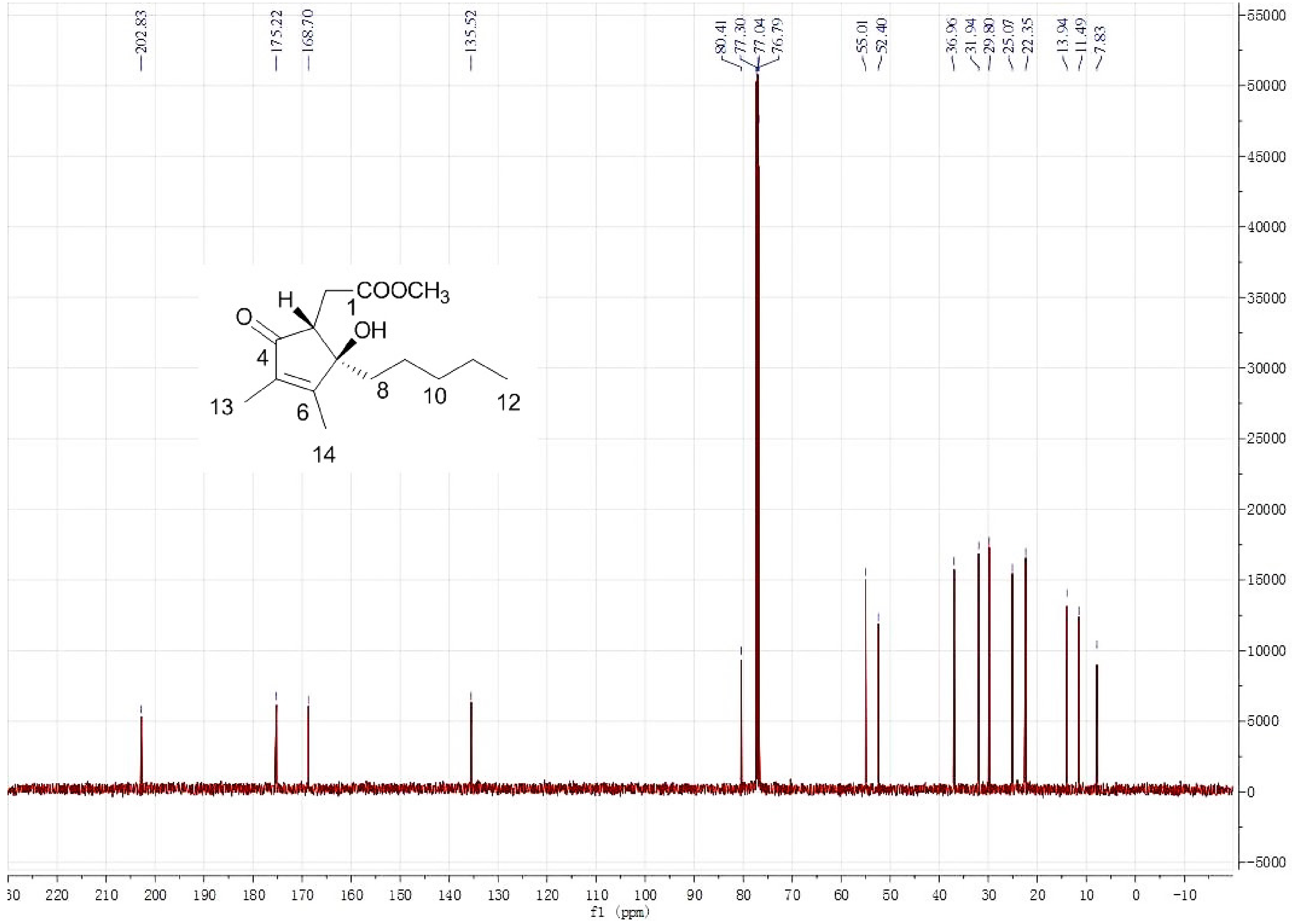

Figure S18. HSQC Spectrum of compound **3** in $CDCl_3$.

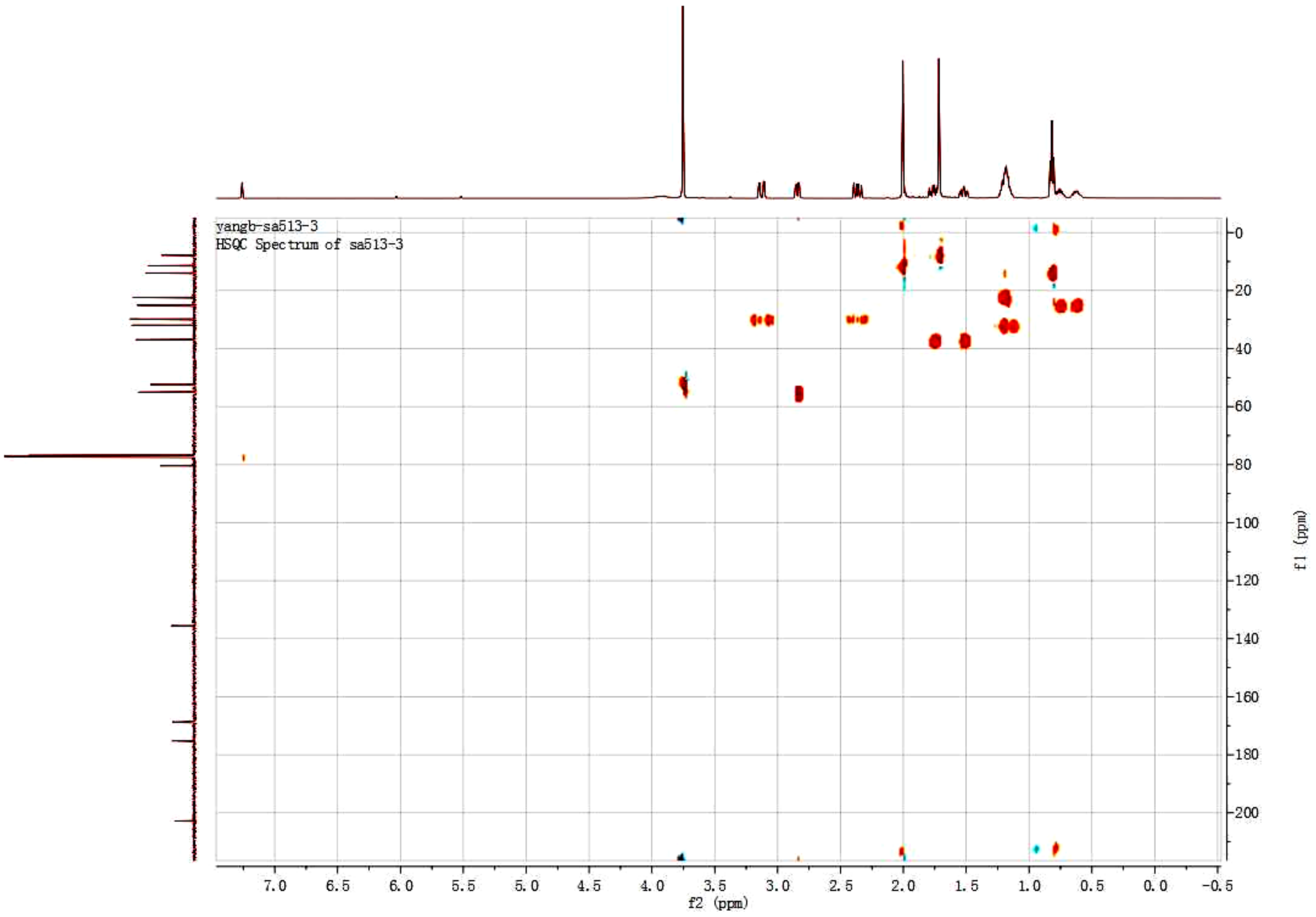

Figure S19. HMBC Spectrum of compound **3** in $CDCl_3$.

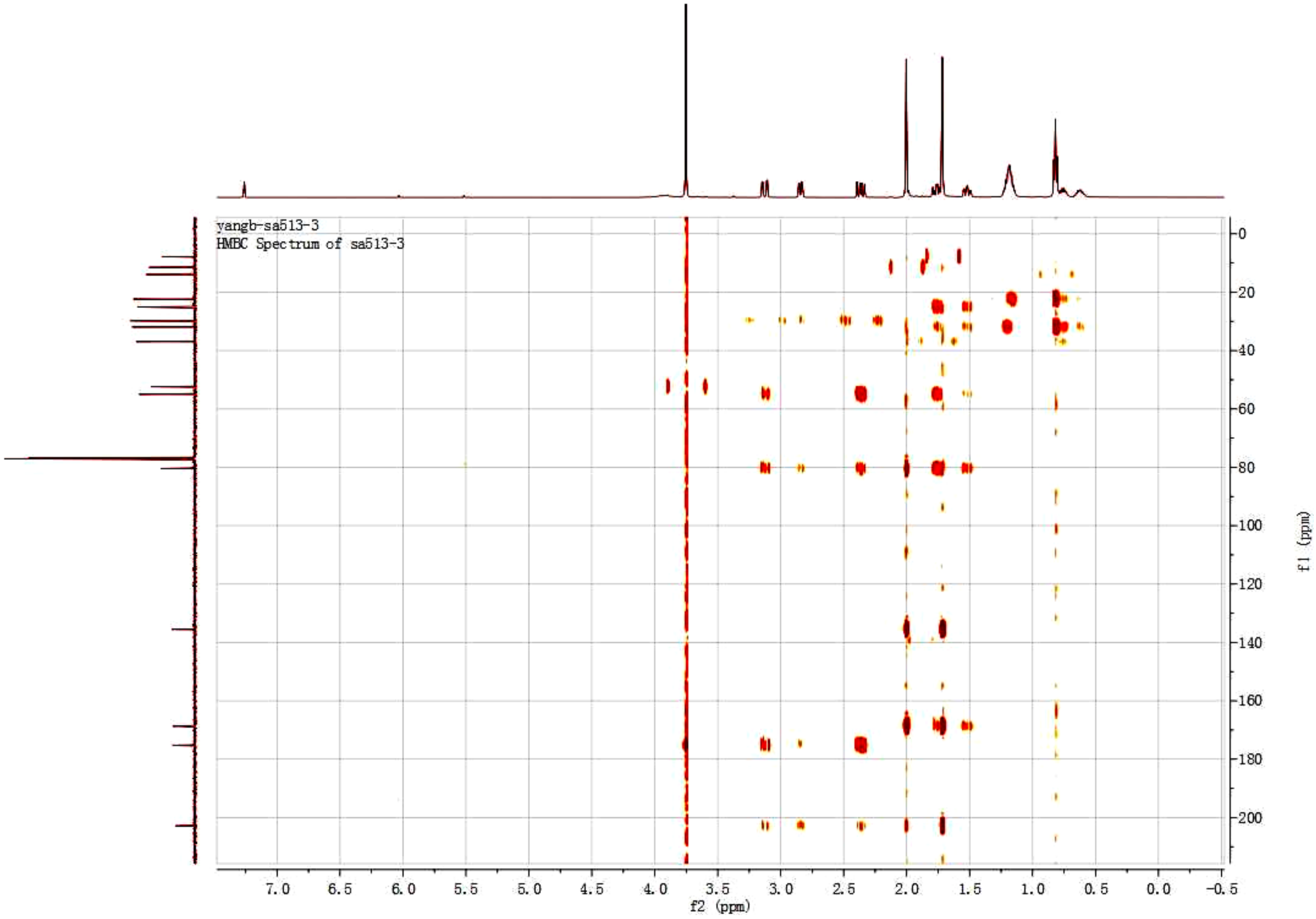

Figure S20. ESIMS spectrum of compound **3**.

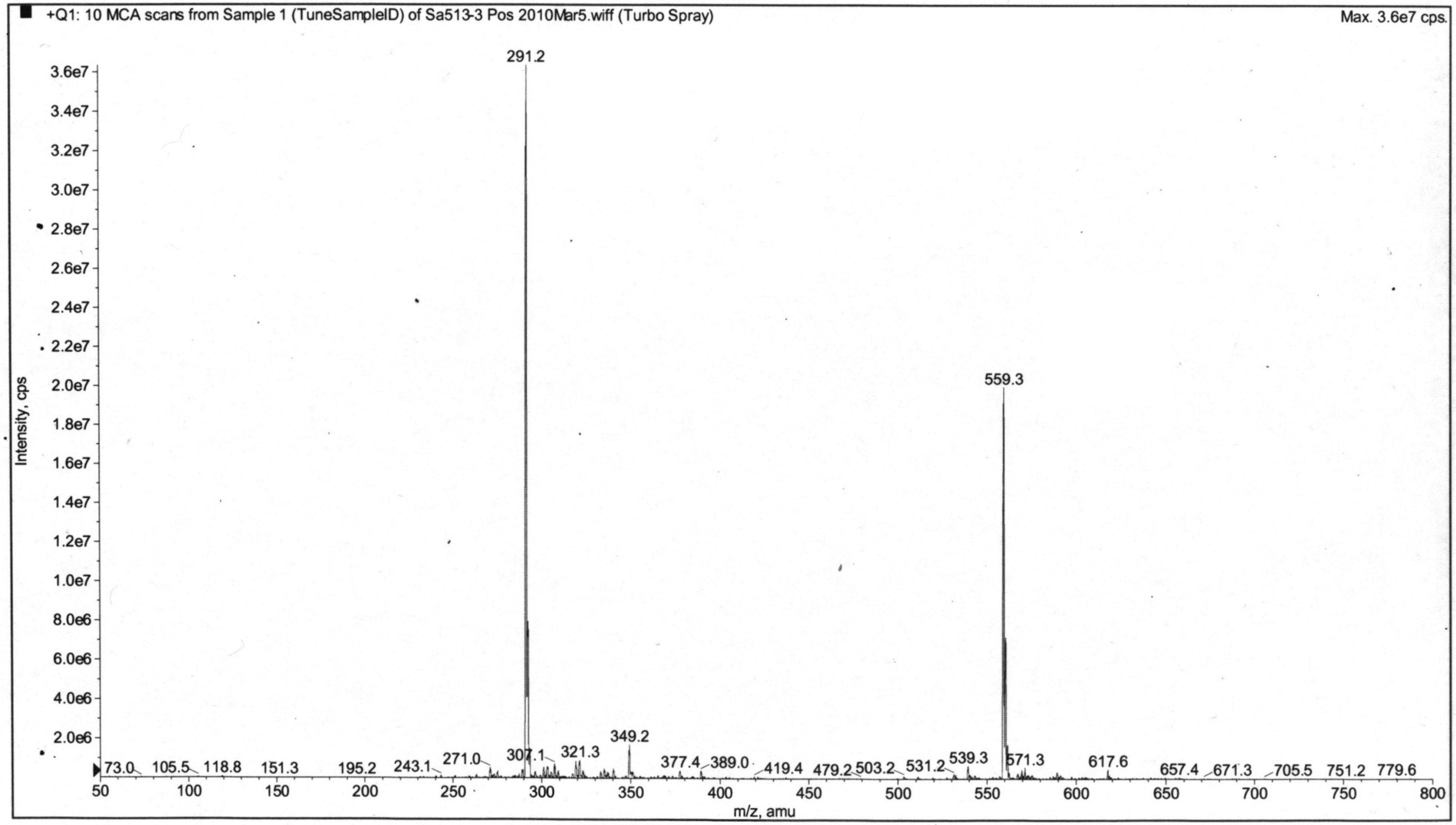

Figure S21. ^{1}H NMR Spectrum (500 MHz) of compound **4** in $CDCl_3$.

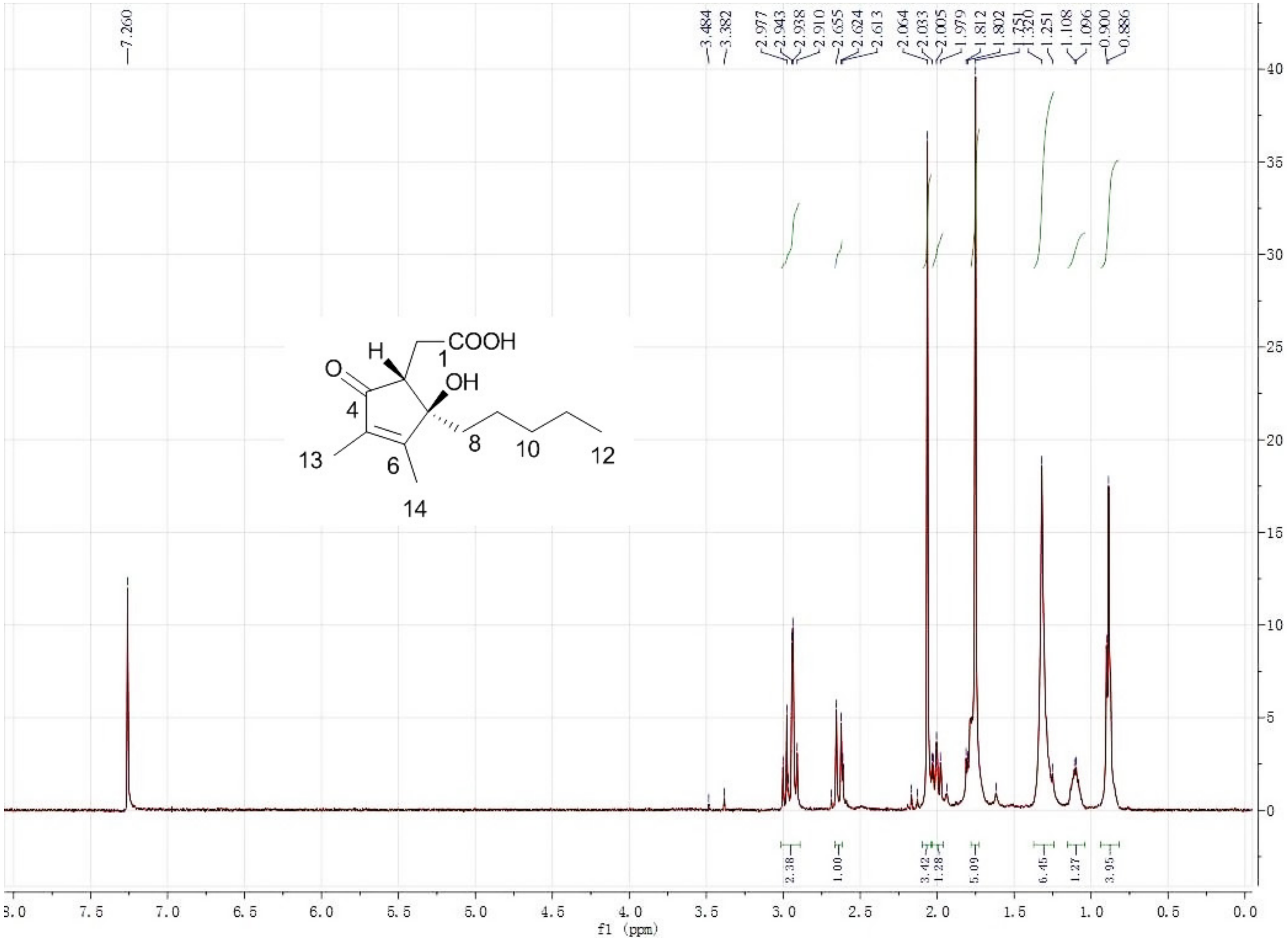

Figure S22. ^{13}C NMR Spectrum (125 MHz) of compound **4** in $CDCl_3$.

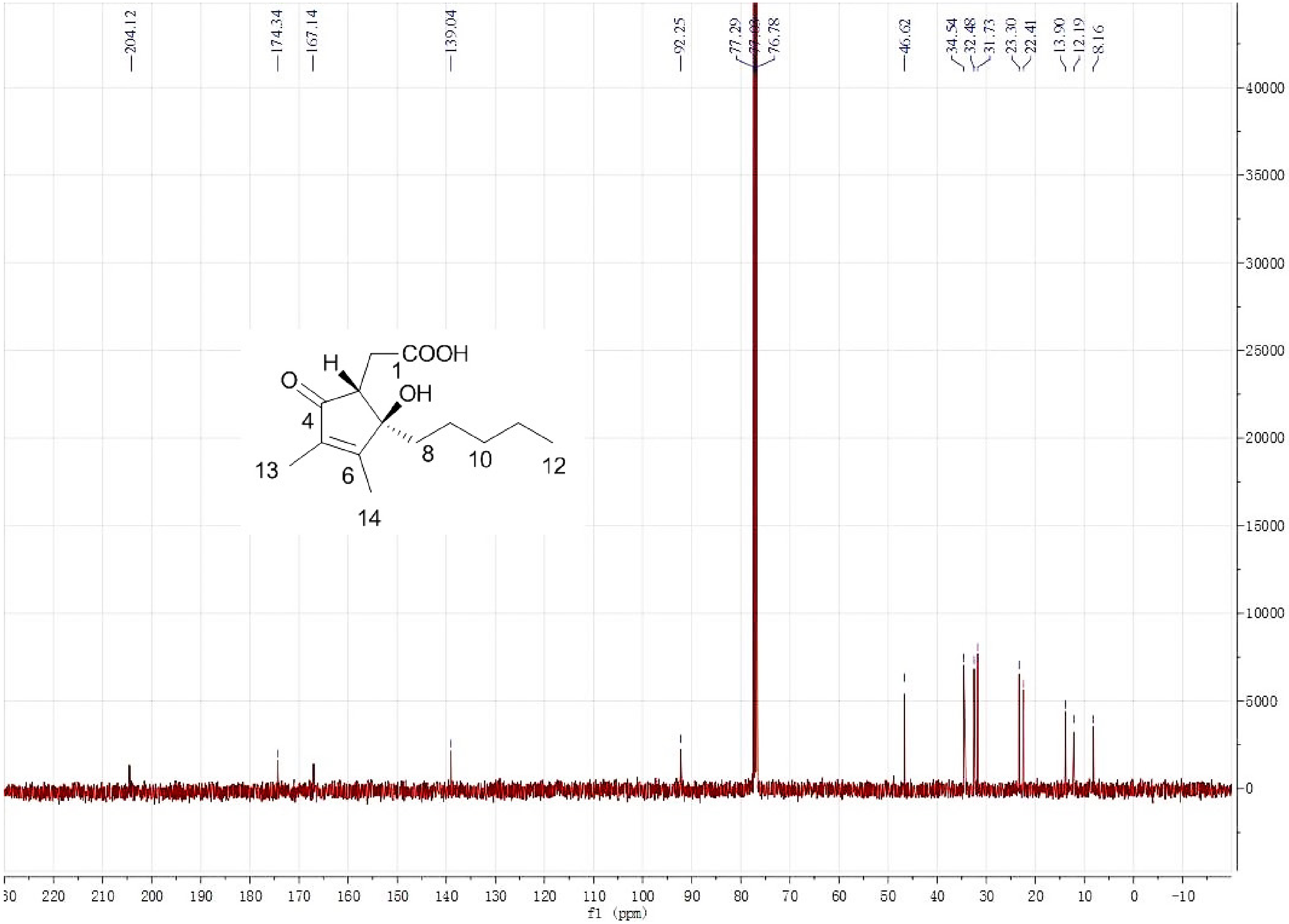

Figure S23. ESIMS spectrum of compound **4**.

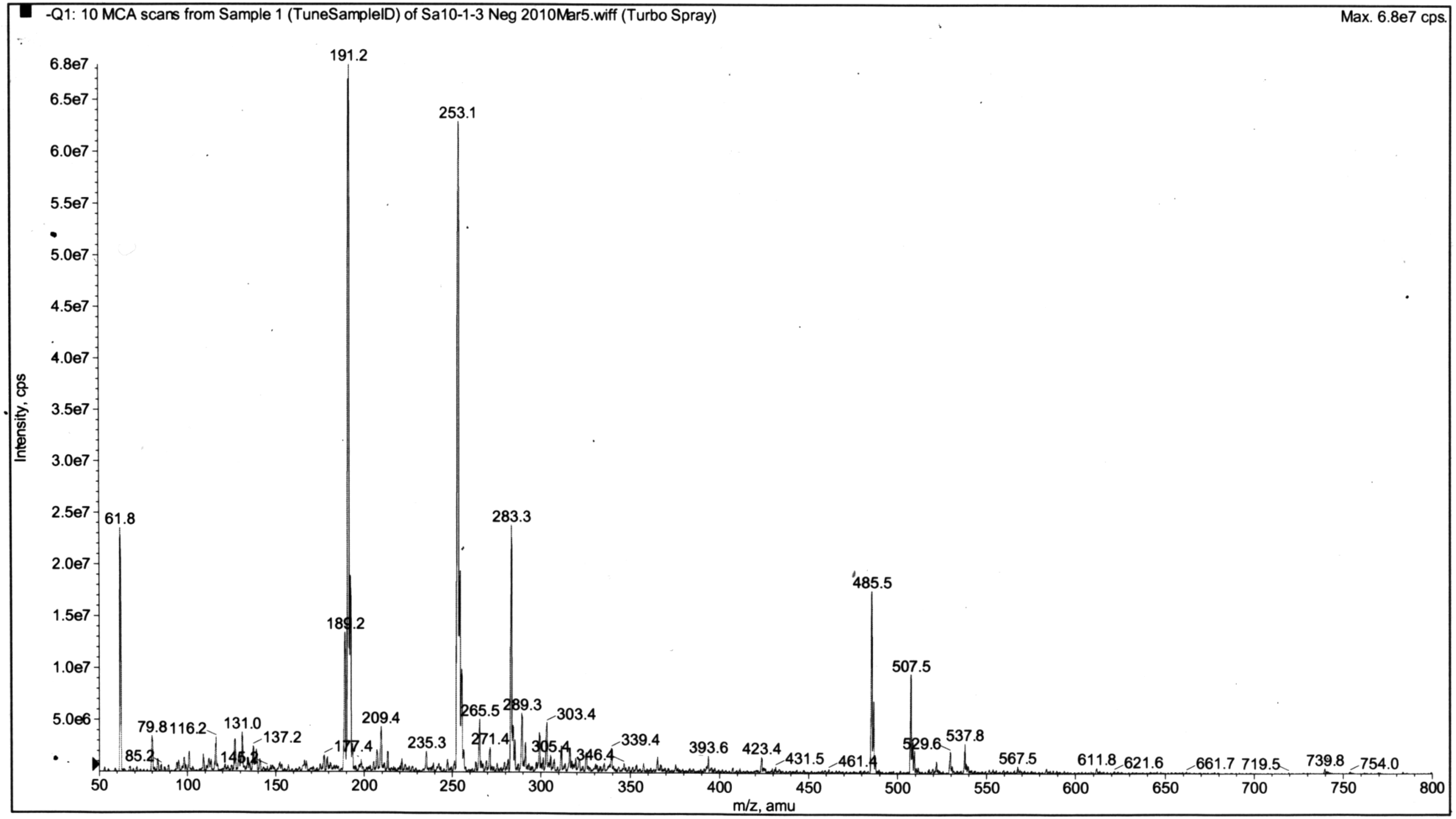

Figure S24. ^{1}H NMR Spectrum (500 MHz) of compound **5** in $CDCl_3$.

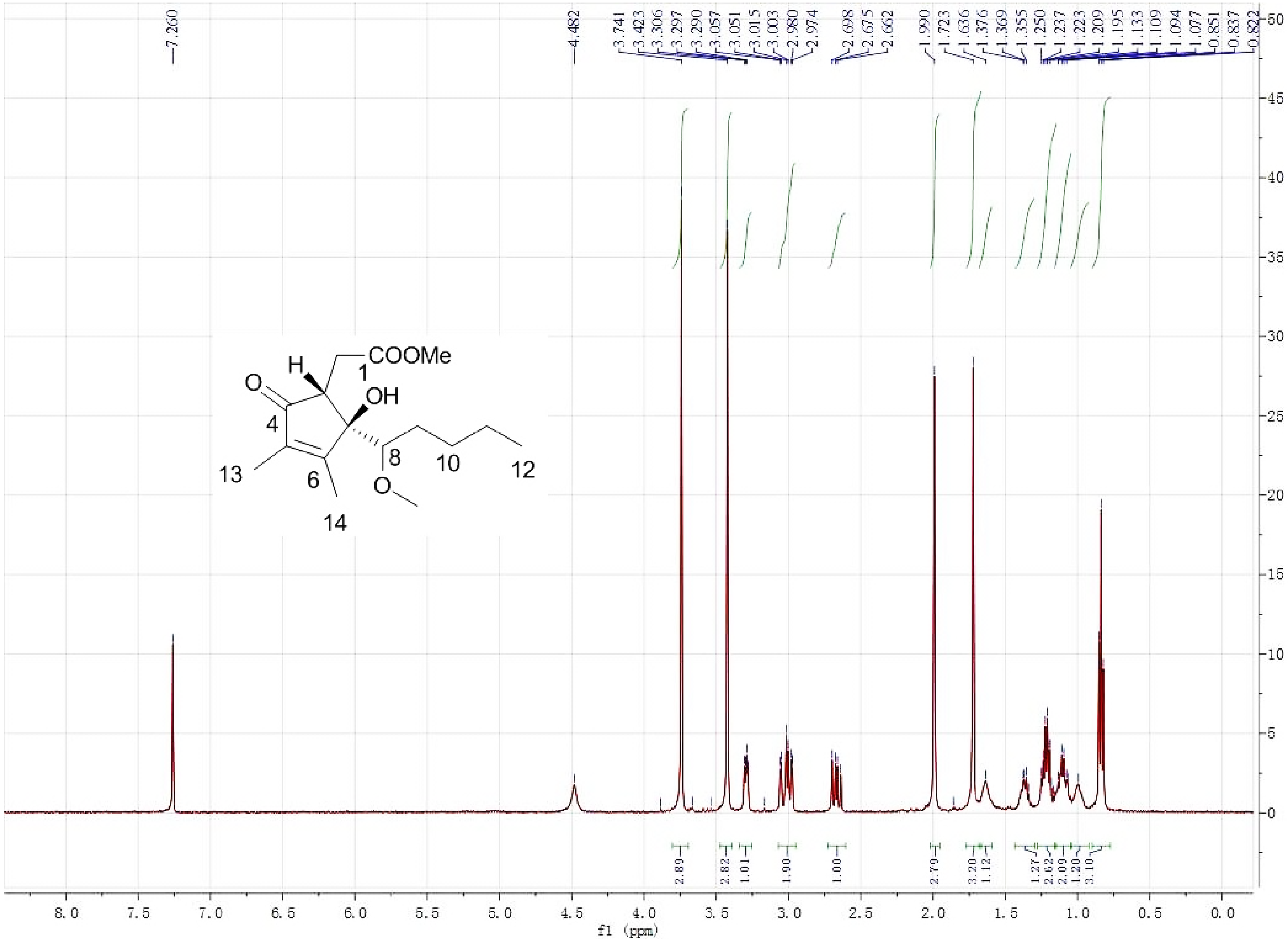

Figure S25. ^{13}C NMR Spectrum (125 MHz) of compound **5** in $CDCl_3$.

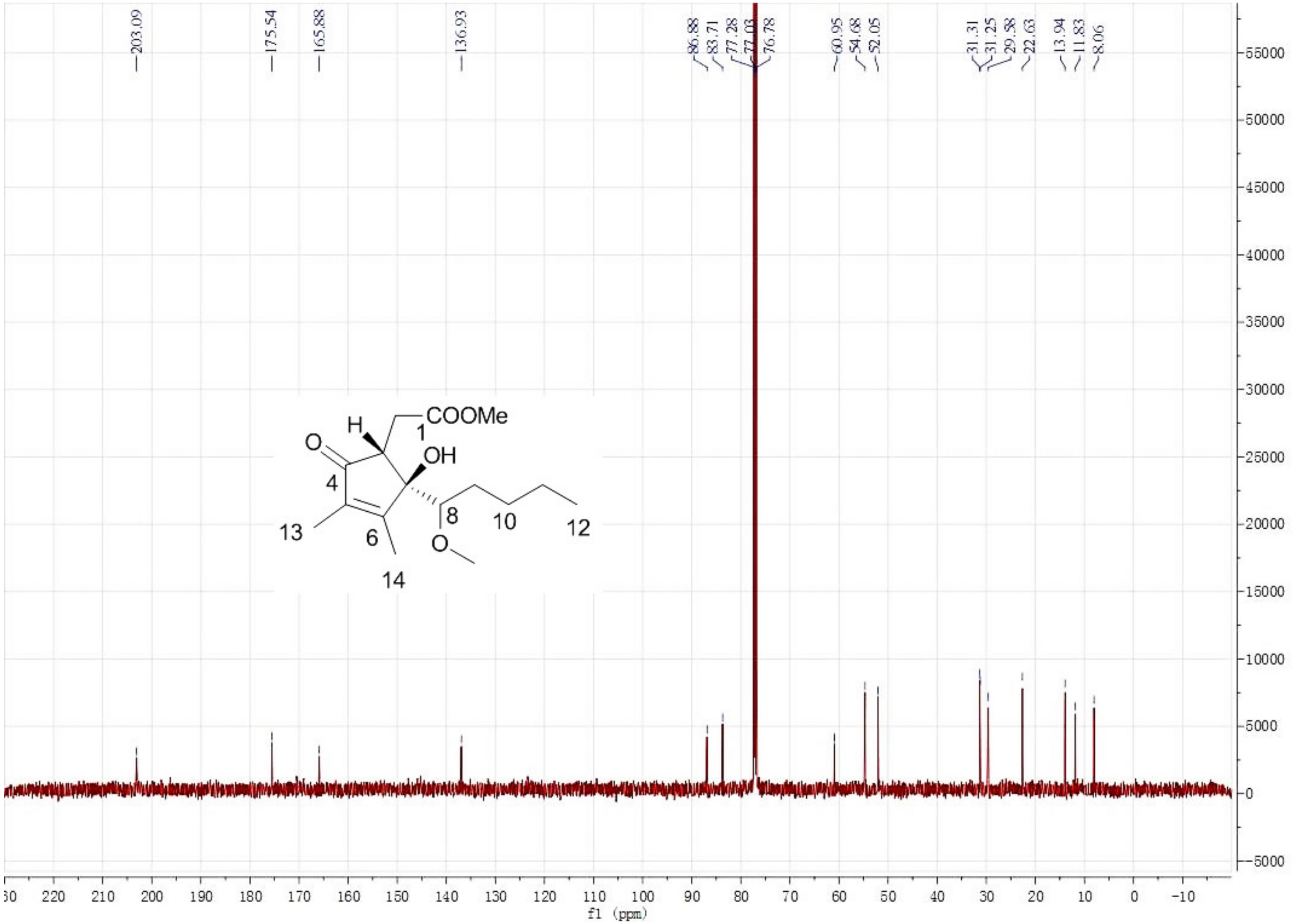

Figure S26. HSQC Spectrum of compound **5** in $CDCl_3$.

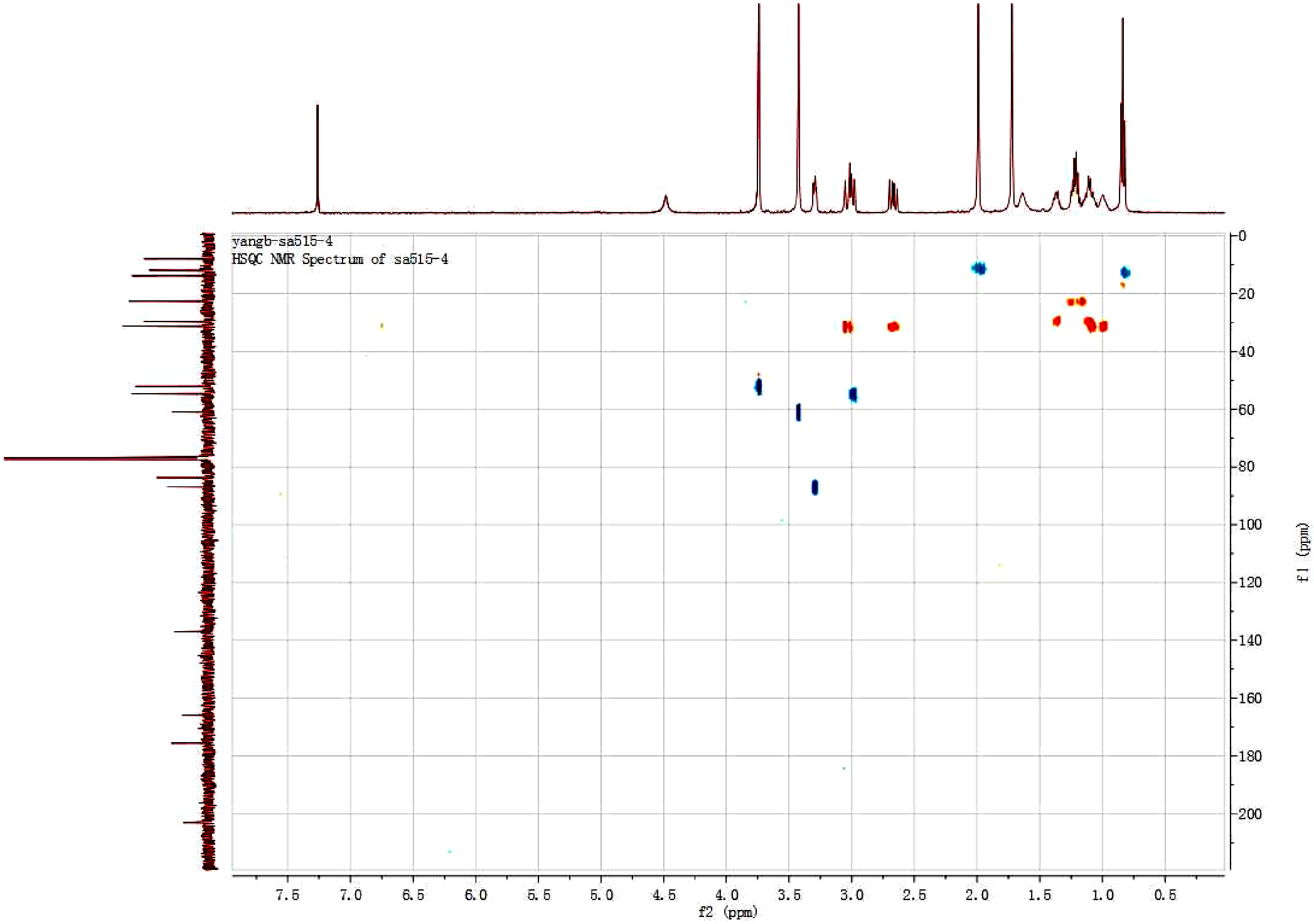

Figure S27. HMBC Spectrum of compound **5** in $CDCl_3$.

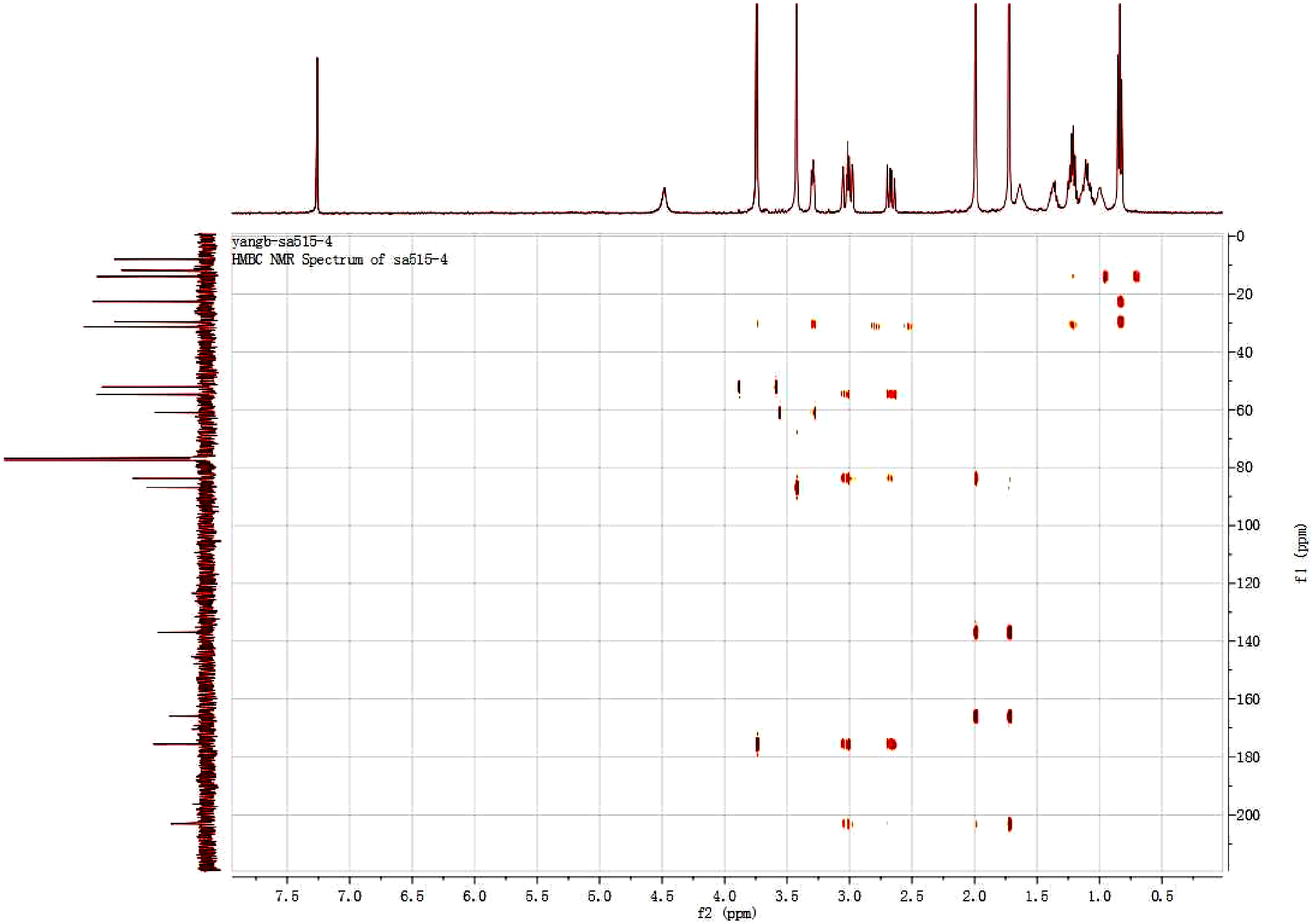

Figure S28. ESIMS spectrum of compound **5**.

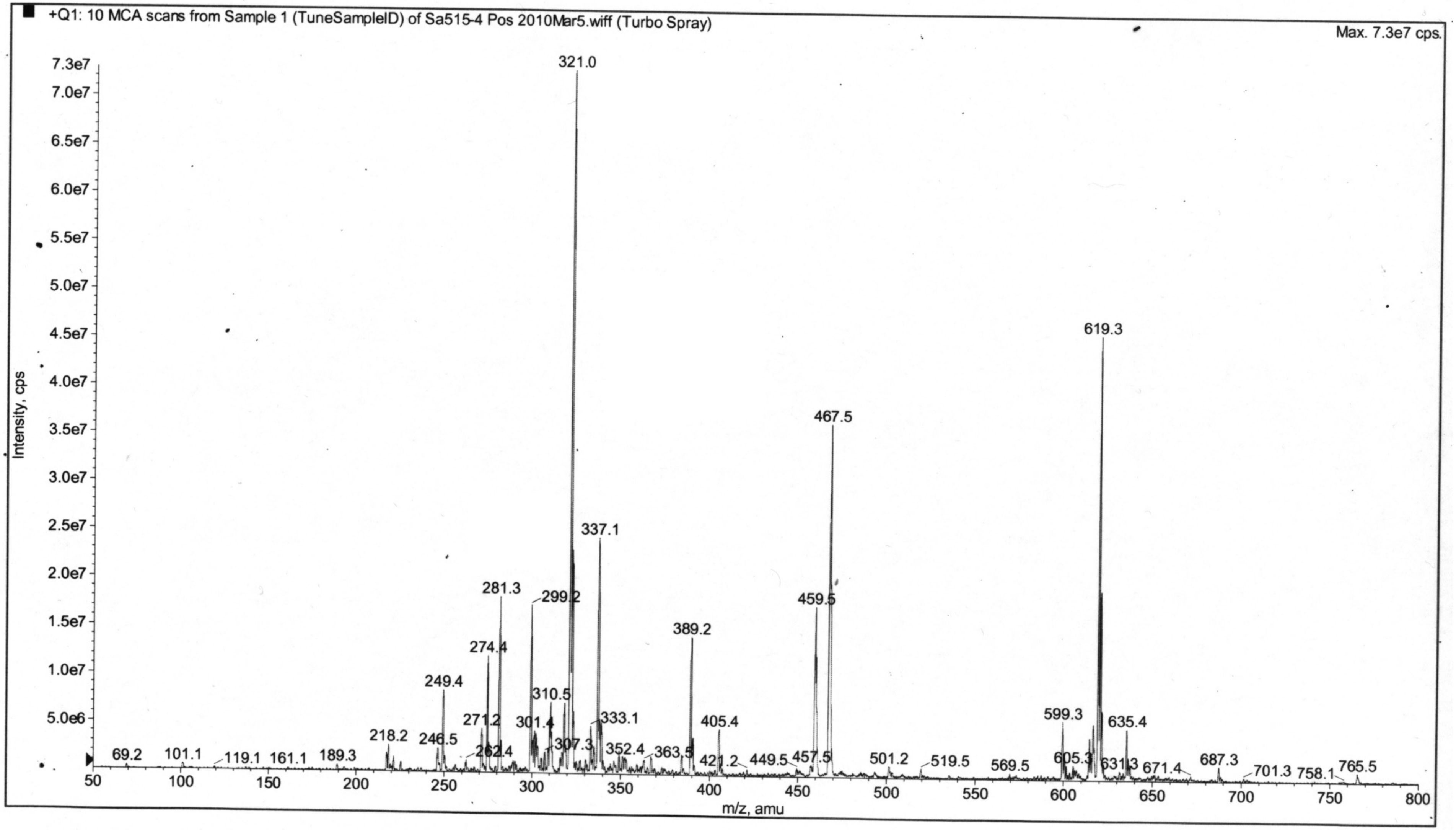

Figure S29. ^{1}H NMR Spectrum (500 MHz) of compound **6** in $CDCl_3$.

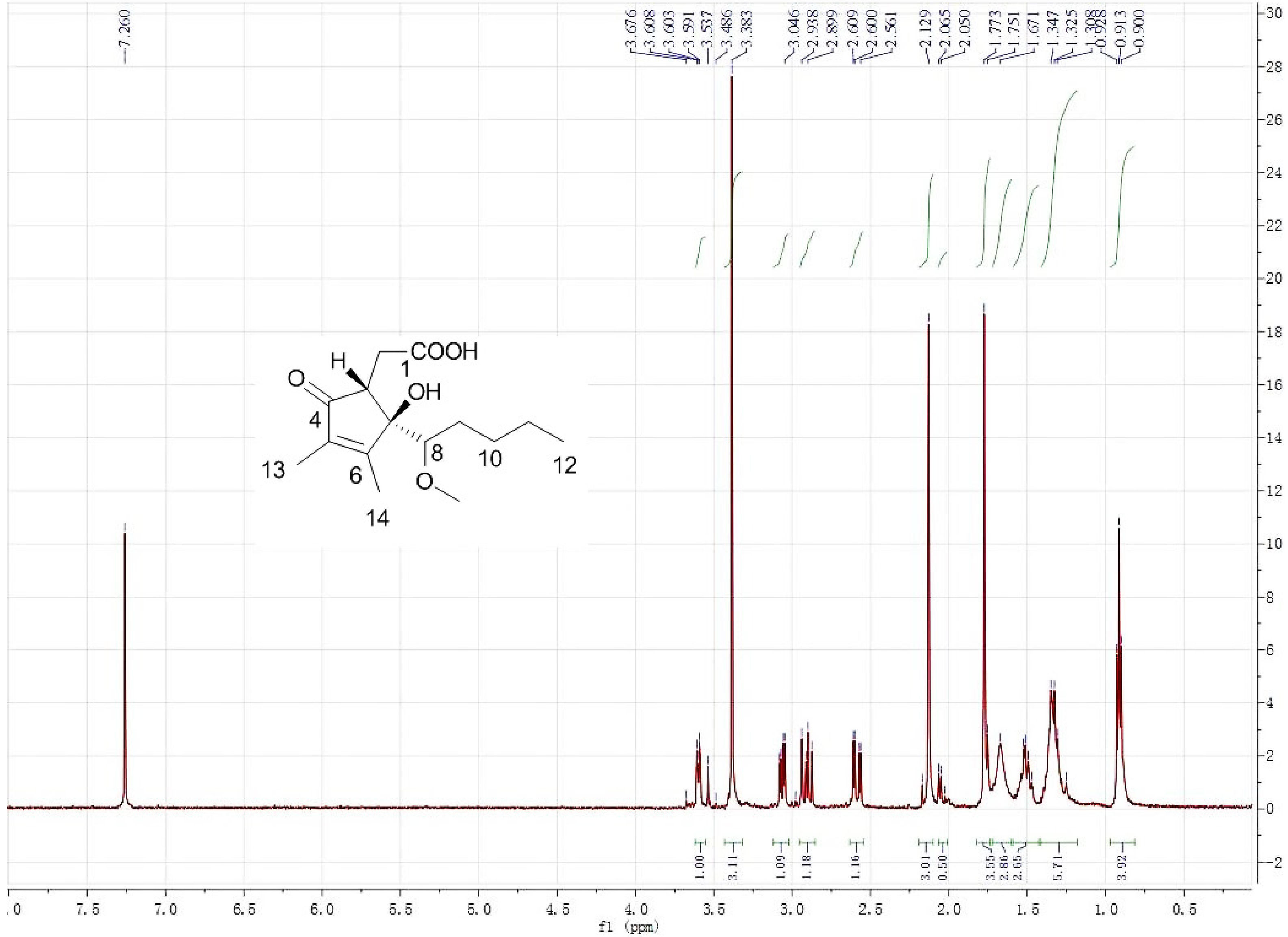

Figure S30. ^{13}C NMR Spectrum (125 MHz) of compound **6** in $CDCl_3$.

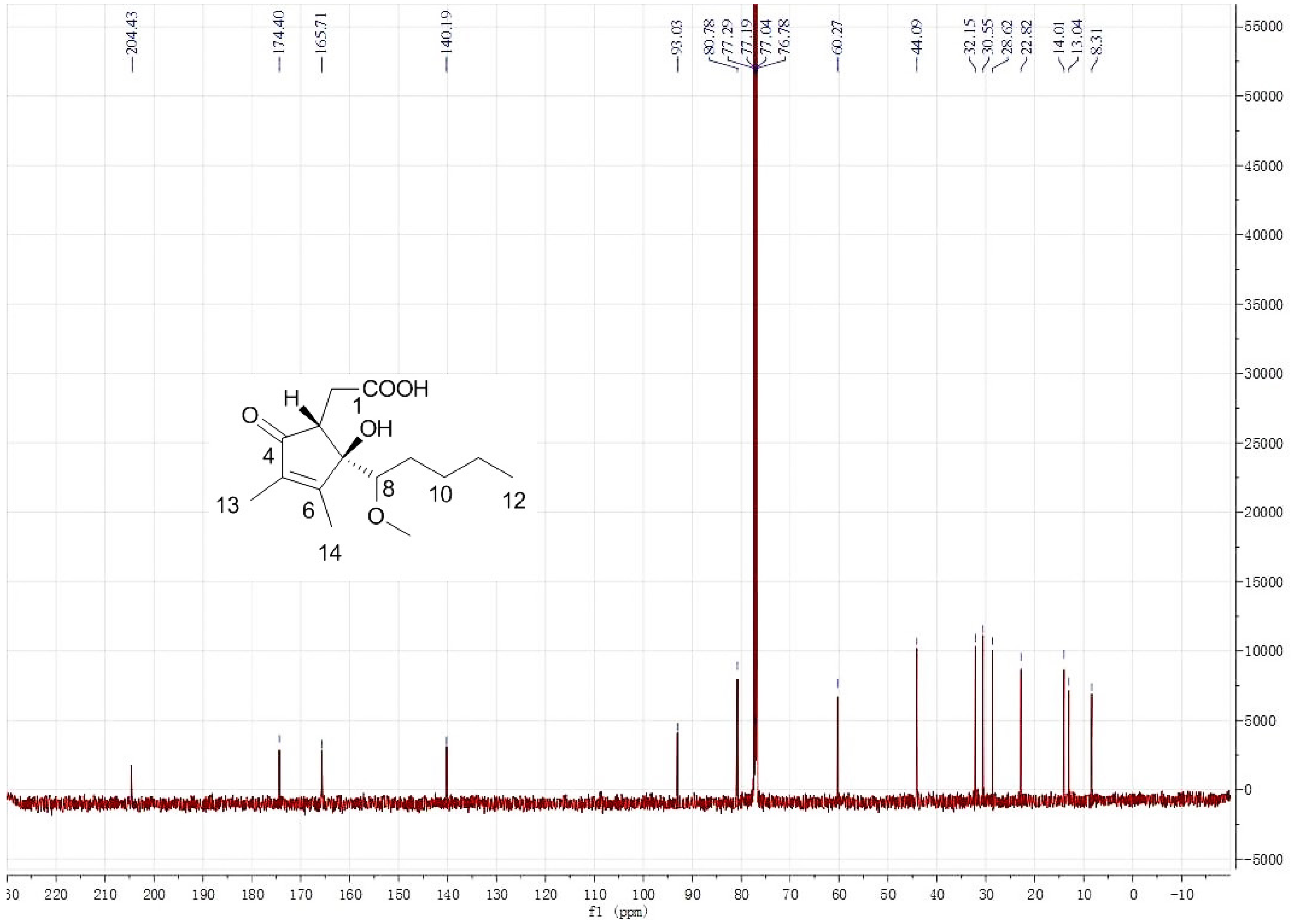

Figure S31. ESIMS spectrum of compound **6**.

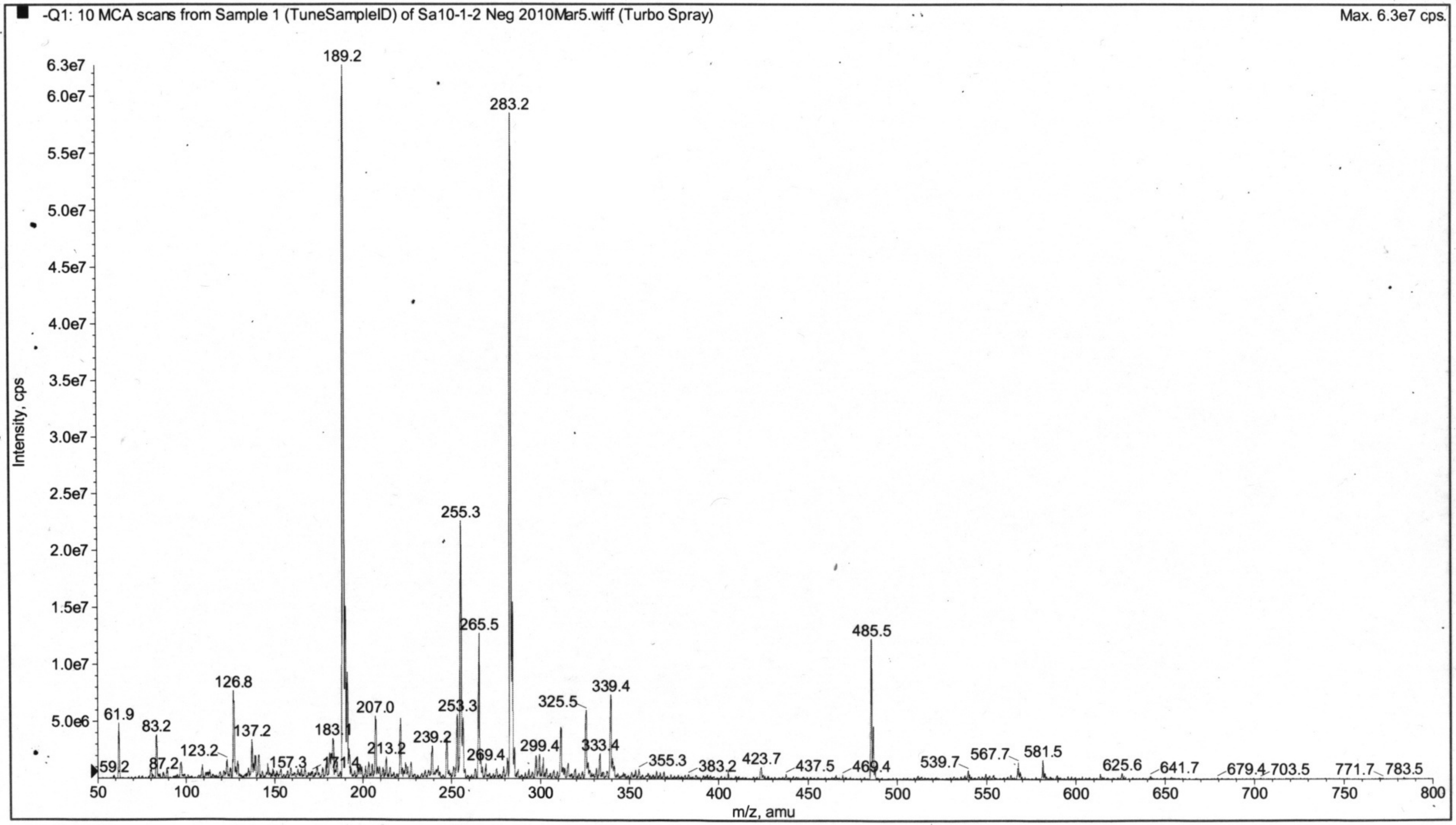

Figure S32. ^{1}H NMR Spectrum (500 MHz) of compound **7** in $CDCl_3$.

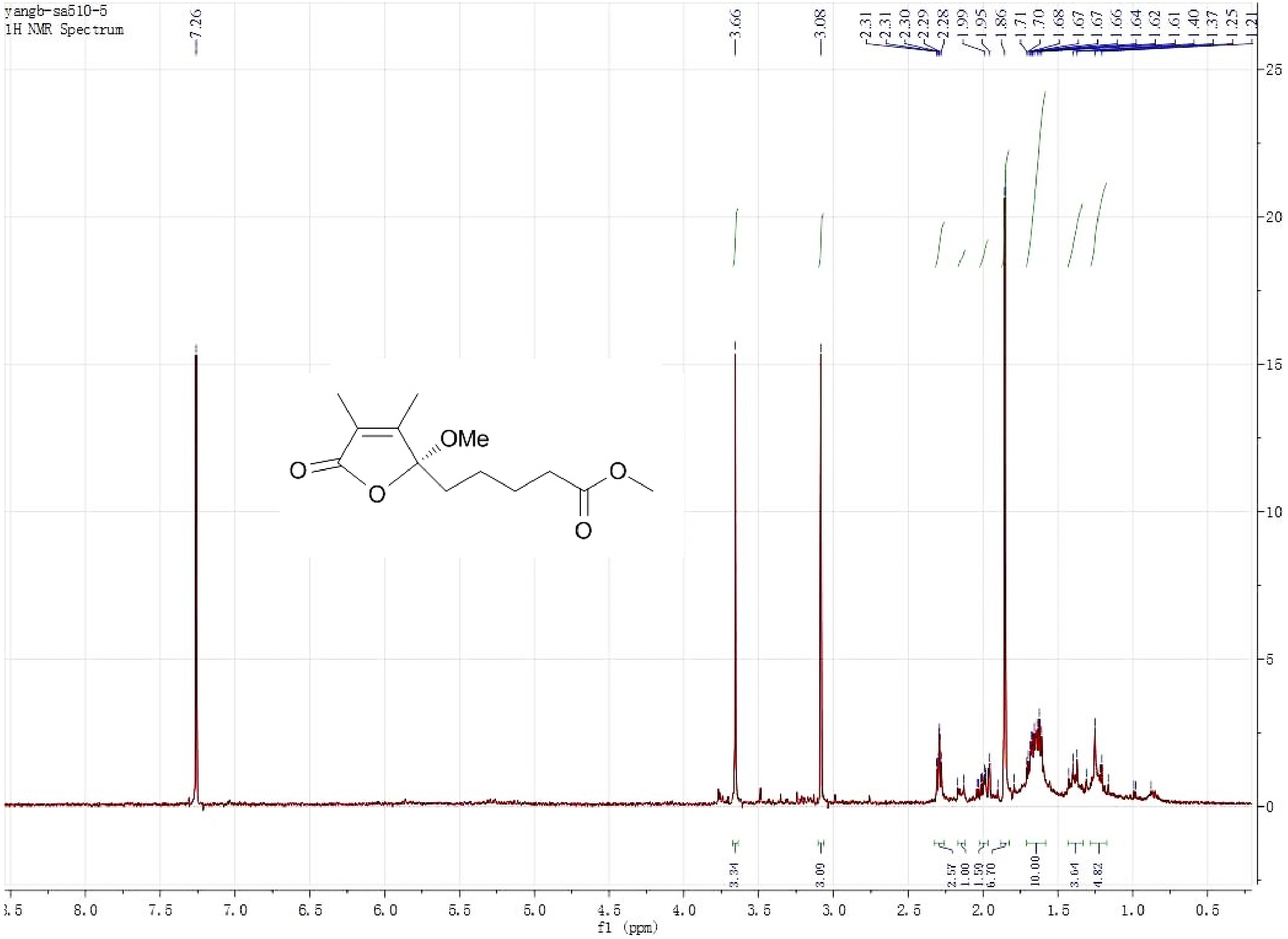

Figure S33. ^{13}C NMR Spectrum (125 MHz) of compound**7** in $CDCl_3$.

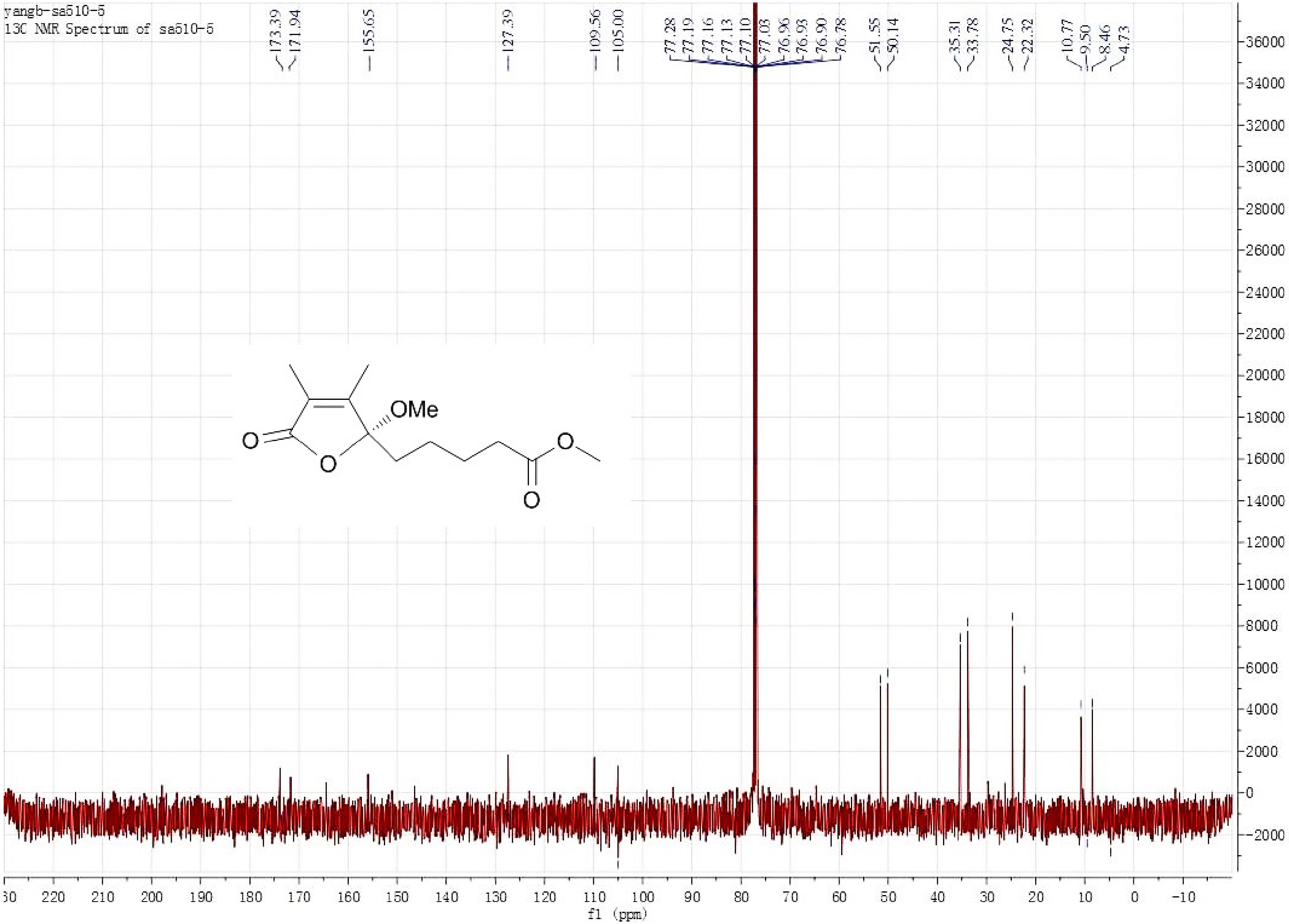

Figure S34. HSQC Spectrum of compound **7**in CDCl3.

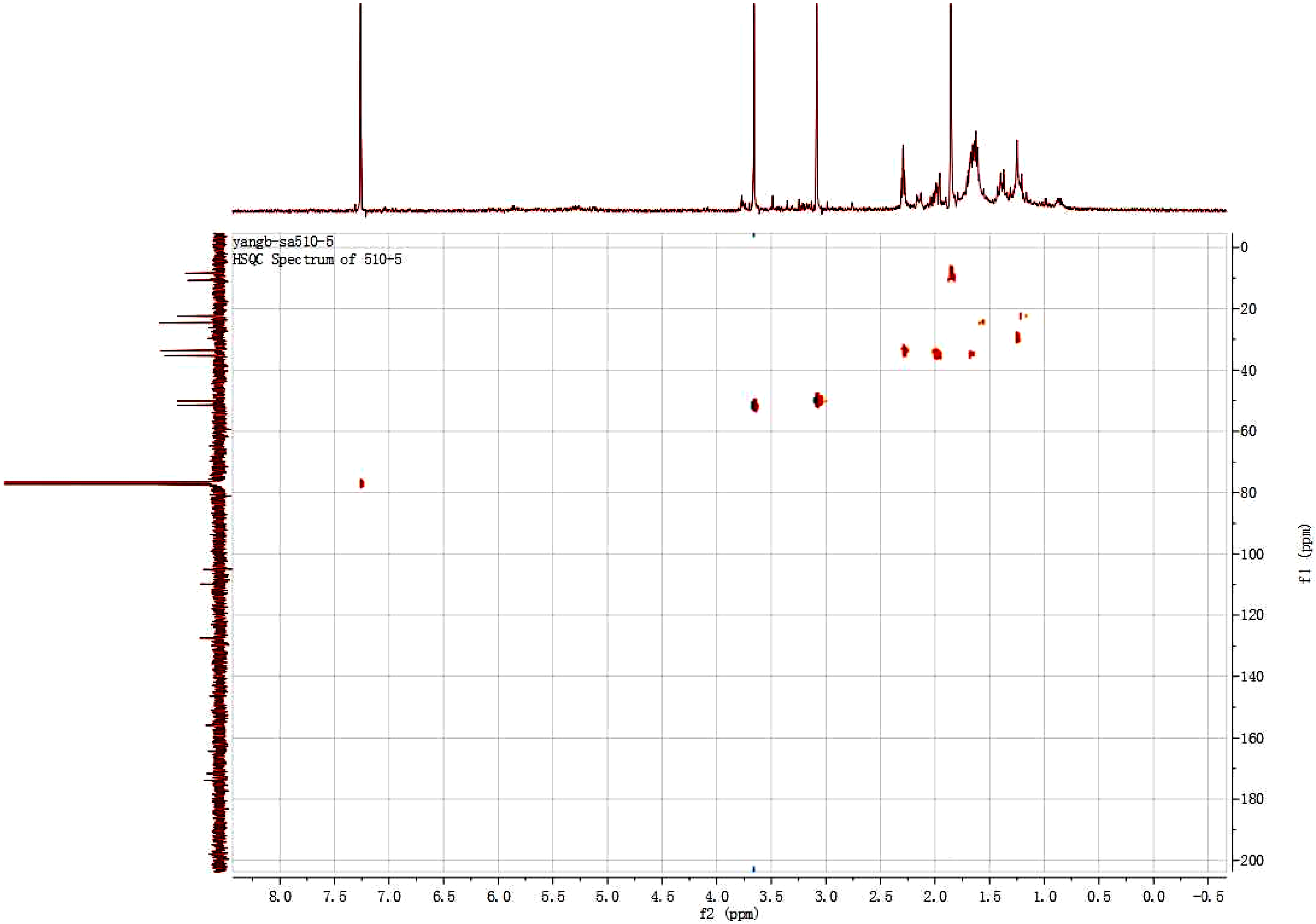

Figure S35. HMBC Spectrum of compound **7**in $CDCl_3$.

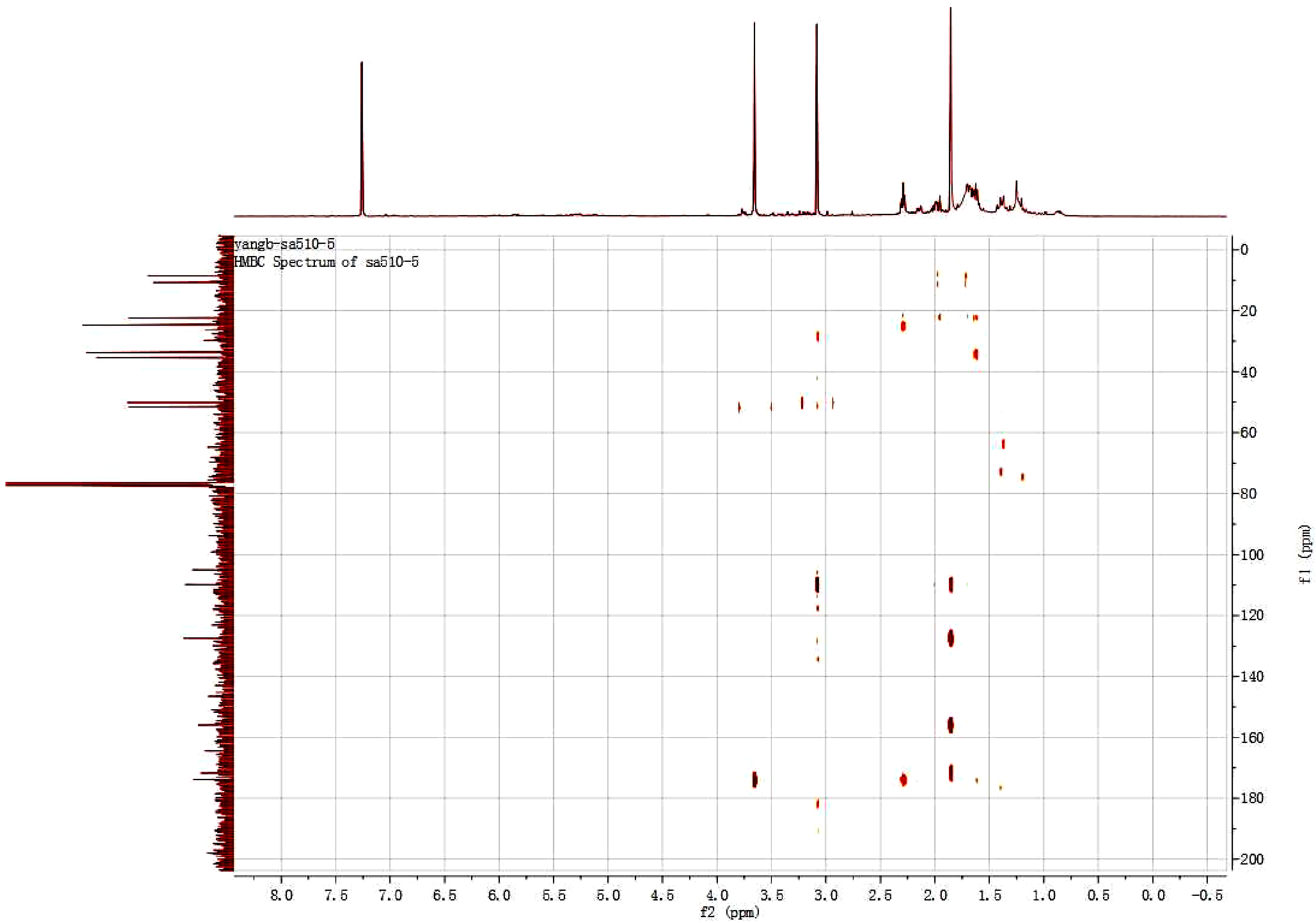

Figure S36. ESIMS spectrum of compound **7**.

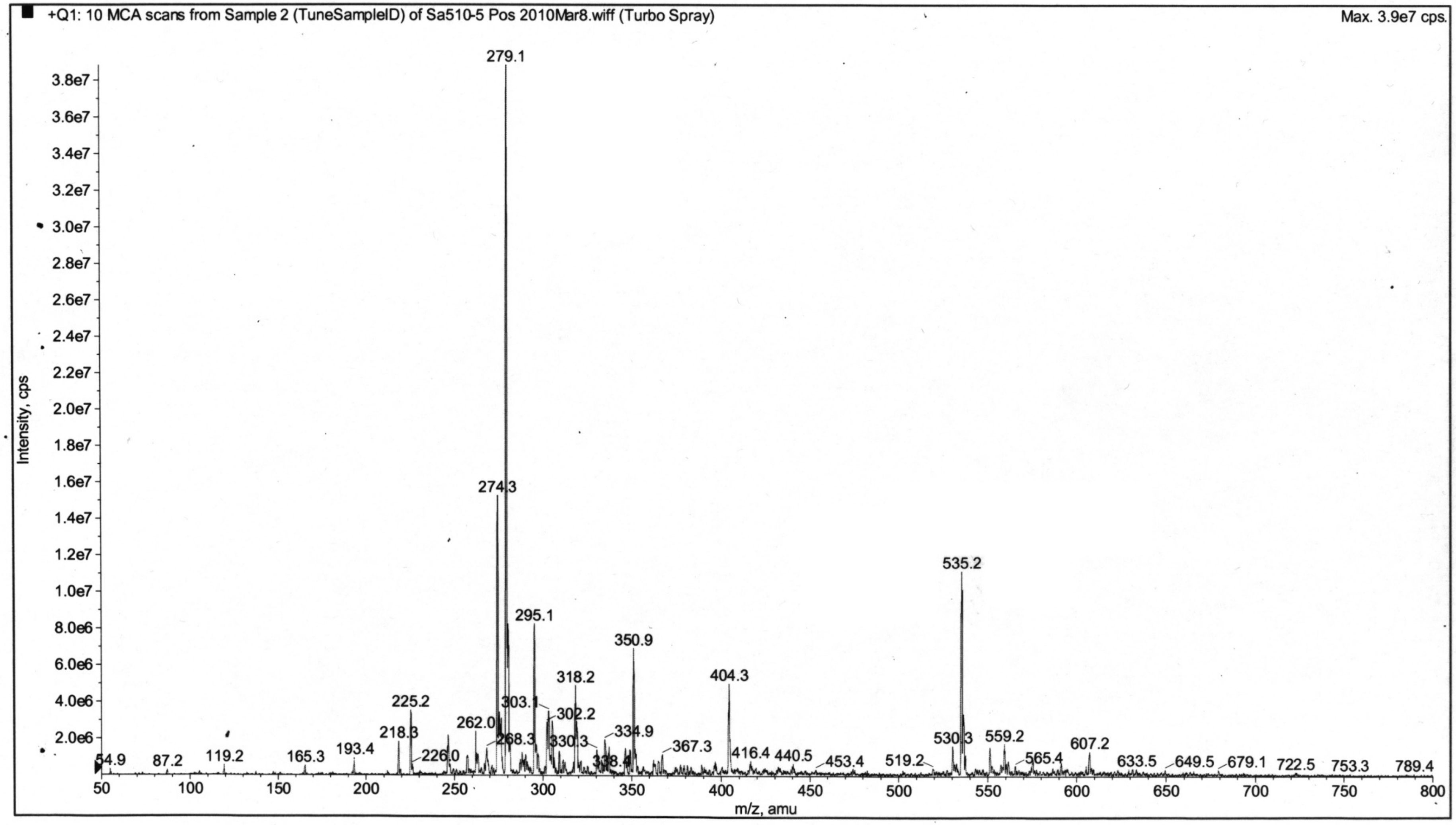

Figure S37. ^{1}H NMR Spectrum (500 MHz) of compound **8** in $CDCl_3$.

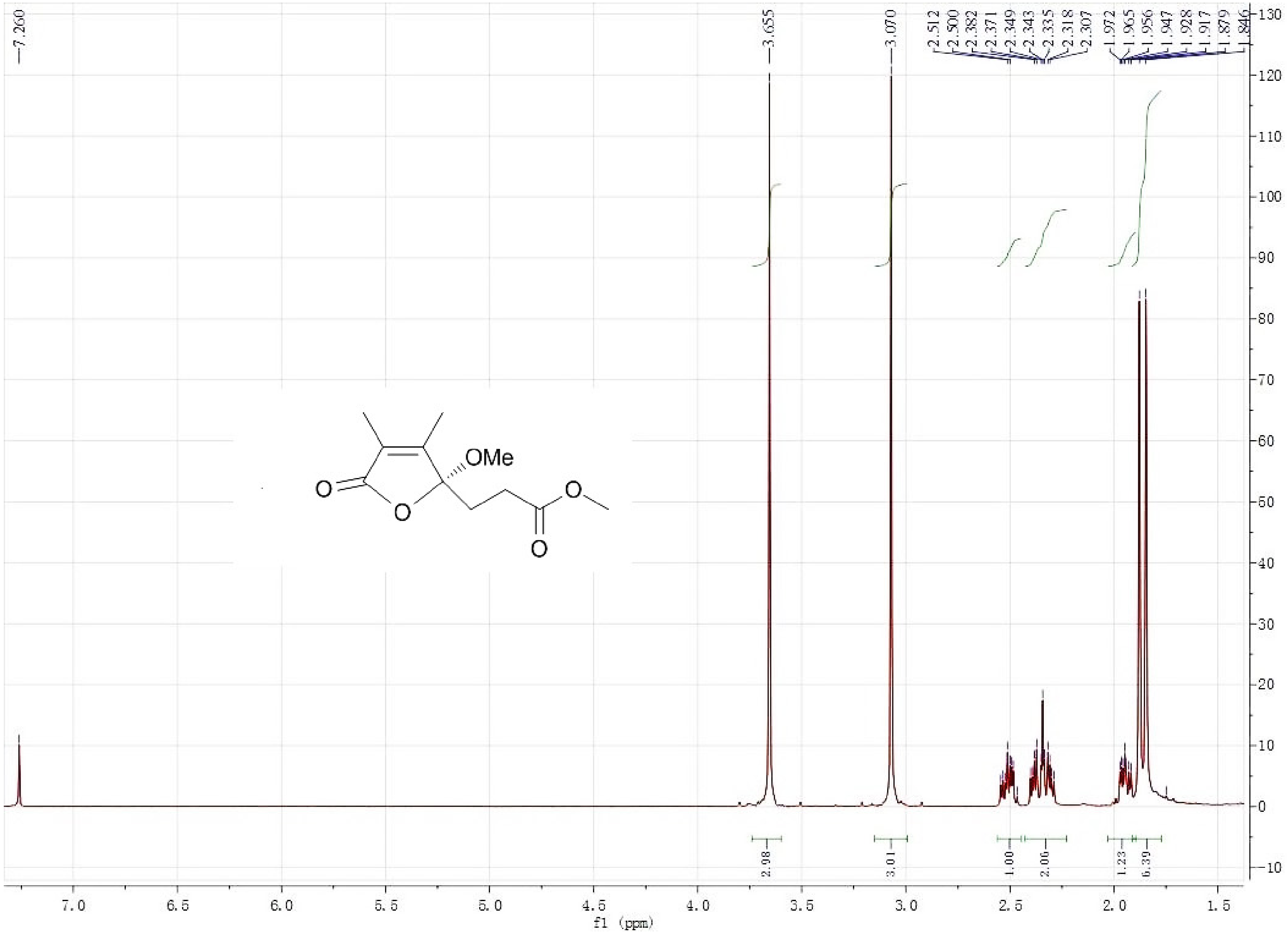

Figure S38. ^{13}C NMR Spectrum (125 MHz) of compound **8** in $CDCl_3$.

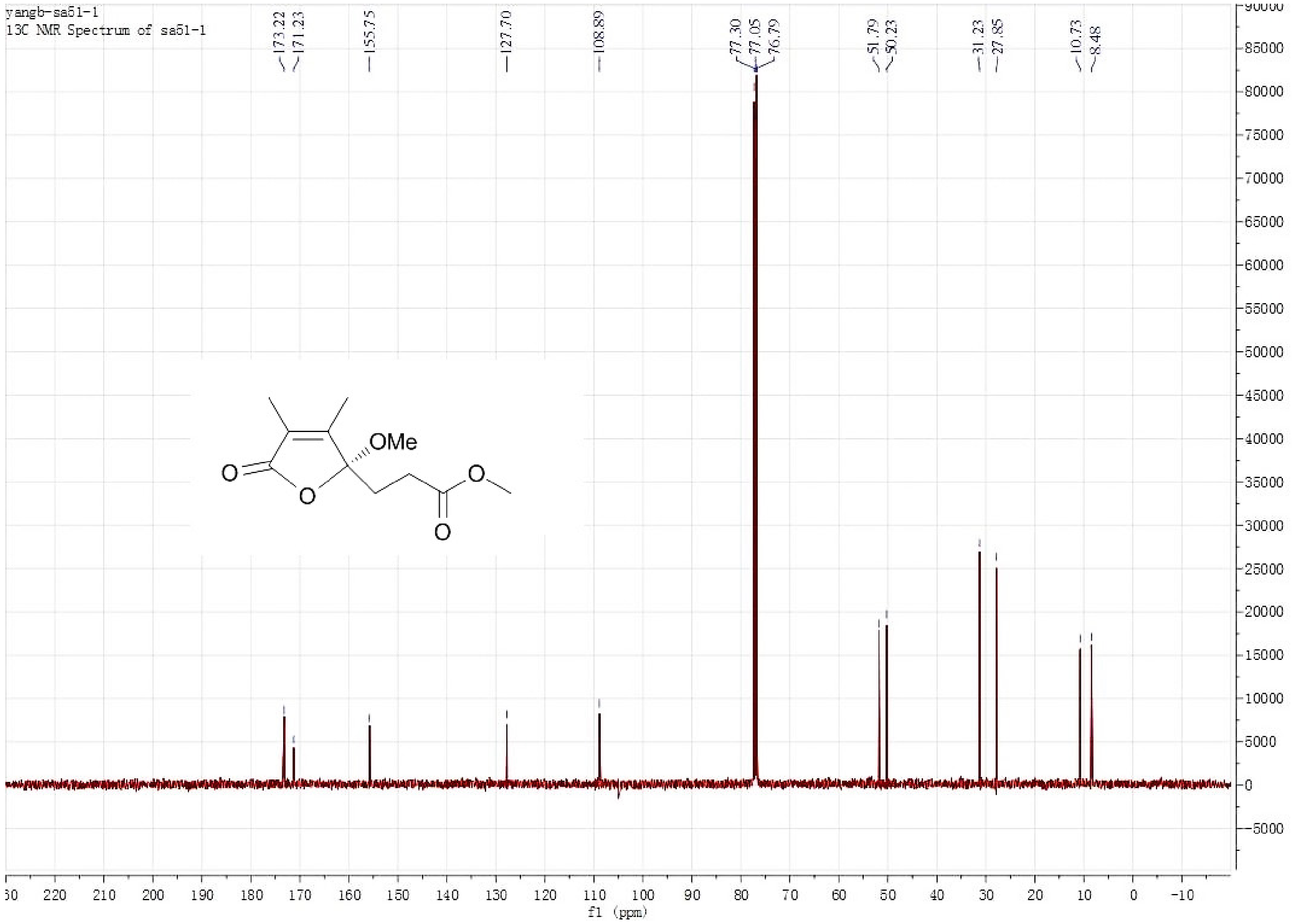

Figure S39. ESIMS spectrum of compound **8**.

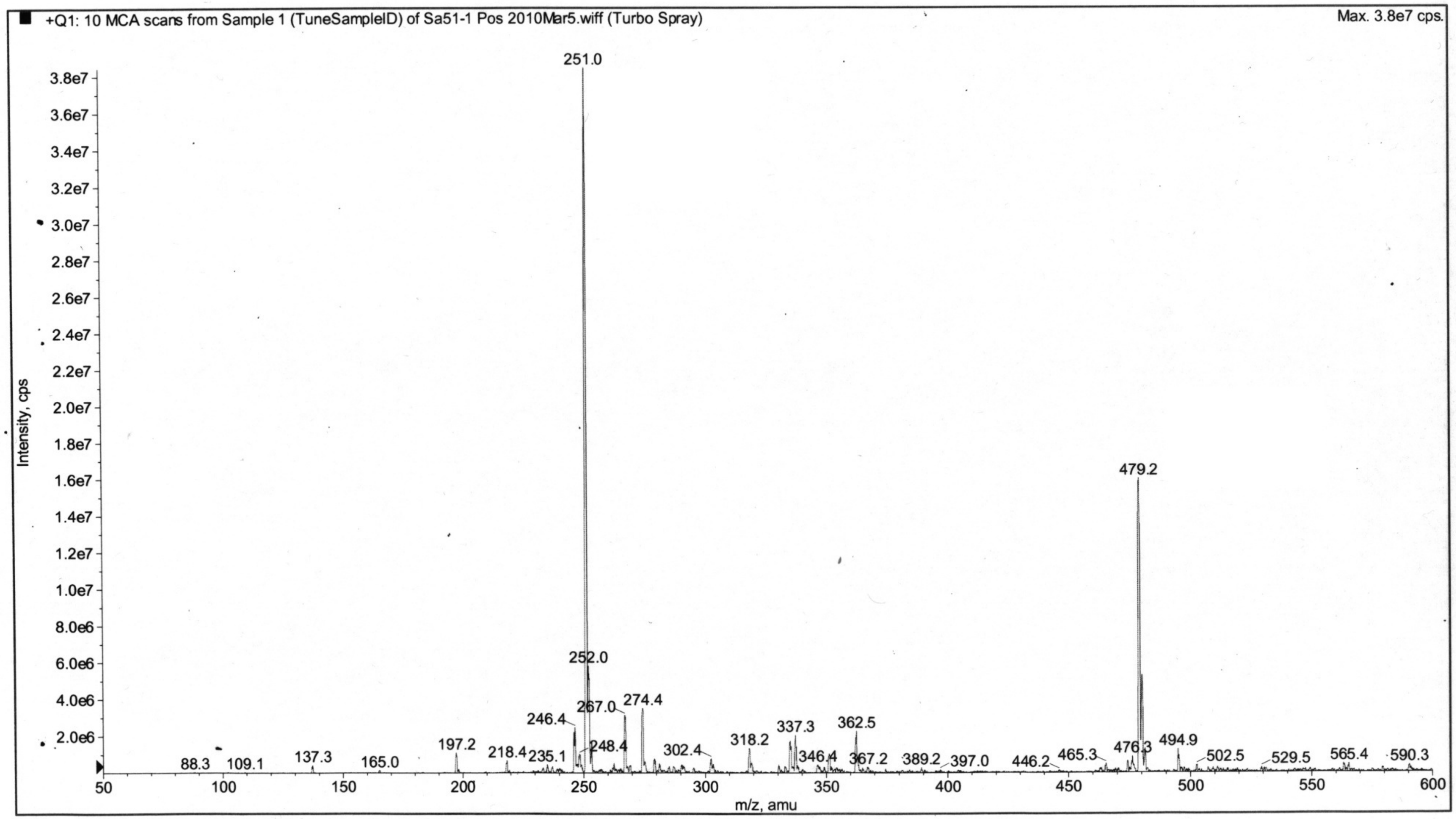

© 2014 by the authors; licensee MDPI, Basel, Switzerland. This article is an open access article distributed under the terms and conditions of the Creative Commons Attribution license (http://creativecommons.org/licenses/by/4.0/).
